# QoS improvement with an optimum controller selection for software-defined networks

**DOI:** 10.1371/journal.pone.0217631

**Published:** 2019-05-31

**Authors:** Jehad Ali, Byeong-hee Roh, Seungwoon Lee

**Affiliations:** Computer Engineering Department, Ajou University, Suwon-si, South Korea; Chongqing University of Posts and Telecommunications, CHINA

## Abstract

The software-defined networking (SDN) paradigm has simplified the management of computer networks by decoupling data and control planes. Moreover, the separation of the data and control planes has transitioned network complexity from traditional devices to controllers; therefore, controllers have become indispensable entities in SDN. Controllers have multiple features and direct the network from a central point and respond to updates to topological changes. However, the supportive capability of these features is strong in one controller but weak in another. Due to several controllers and each controller having a set of features, selecting an optimal SDN controller can be considered to be a multi-criteria decision-making (MCDM) problem. Herein, a two-step approach is proposed for SDN controller selection. First, the controllers are ranked with analytical network process (ANP) according to their qualitative features which influence the performance of these controllers and then a performance comparison is performed to check for the QoS improvement. The controller with a high-weight value from the feature-based comparison is quantitatively analysed by experimental analysis. The main contribution of this paper is checking the applicability of the ANP for controller selection in SDN considering its features and performance analysis in real-world Internet and Brite topologies. The simulation results show that the controller computed through the proposed approach outperforms the controller selected with existing approaches. The selection of an optimum controller with ANP results in a reduction of topology discovery time and delay in the normal and traffic load scenario. Similarly, an increase in throughput with a reasonable utilization of the central processing unit (CPU) is observed for the proposed controller.

## Introduction

The Contemporary computer networks have been revolutionized owing to the ease of programmability, innovation, flexibility, and centralized management spearheading concepts broached by SDN. Its abstraction has reduced the complexity of traditional network devices by shifting the distributed control logic from these devices to a central controller, i.e., separating data and control planes. The data flow is controlled by the centralized controller logic along with the applications running and interacting with the controller through its northbound interface and the data plane devices. This control is managed by a protocol known as OpenFlow [1].

The innovative features of flexibility management and the support for network function virtualization (NFV) [2] broached by SDN have made it an exquisite choice for the Internet of Things (IoT) and 5G. Similarly, megacorp IT firms such as Google, Amazon, and Facebook have already adopted SDN and are contributing to the Open Networking Foundation (ONF) [3] for the standardization of SDN protocols and architecture. A well-known project called B4 [4] was initiated by Google to connect its international data centers by leveraging innovative SDN features. This allowed its hardware to no longer be vendor dependent, provided centralized management, and created flexibility in network management through well-defined application programming interfaces (APIs) and the virtualization of network functions. All of this has resulted in a reduction in cost, increased efficiency, and fast deployment of new services.

The novel centralized management paradigm introduced by SDN has resulted in an escalation in the importance of the SDN controller. The SDN controller presents a global view of the network under its obligation; therefore, operators can program the data plane devices and apply the policies from a central point in contrast to the distributed and classical internet protocol (IP) networks. However, aside from the many advantages of SDN, its modelling, evaluation, and testing present several challenges. One of the challenges is the selection of an optimum SDN controller, as every controller has multiple supporting features. The obligatory role of the controller in a standard SDN stipulates the selection of an optimum controller.

The comparative study of SDN controllers has been the focus of many studies due to the significant role of the controller. These studies select a controller among a set of controllers based on the performance of the SDN controllers using Cbench [5] or Mininet [6]. The study presented in [7] compared five SDN controllers, NOX, POX, RYU, BEACON, and FLOODLIGHT [8–12], to increase the number of threads and switches in both throughput and latency modes. A similar performance comparison study added two more controllers: MUL and MAESTRO [13–14]. This study can be found in [15], and the authors used Cbench to measure the throughput and latency of each.

The SDN controller performance study in [16] was conducted by first creating a network topology using Mininet and then running it on the controller. Then, the IPERF and PING utilities were utilized to conduct TCP throughput and latency comparisons. The authors compared four controllers, ODL [17], POX, ONOS [18], and RYU, based on their throughput and latency by creating a tree topology with 16 hosts and a fanout of 4. The same performance parameters were considered in [19] using a single topology with one switch and three hosts. In [20], the authors compared the performance of the POX and FLOODLIGHT controllers by using single, linear, and tree topologies.

These works focused on controller selection based on certain performance parameters (throughput and latency) using Cbench or Mininet. However, these studies did not focus any significant attention on the supporting features of these controllers during the selection process neither they have evaluated the performance of these controllers using a real-internet topology. These supporting features can include the following: OpenFlow support, representational state transfer (REST) API, graphical user interface (GUI), Open-stack networking, clustering ability, or modularity and multi-threading. Therefore, the researchers realized the need to consider the features of these controllers and how they contribute to the selection process of an SDN controller.

To the best of our knowledge, this is the first study that evaluates the performance of the controllers selected based on their feature set in the real internet topologies from zoo dataset [21] and Brite [22] topology generator. In this paper, we propose a hybrid approach for SDN controller selection, and we solve our computations in two stages. The first stage performs controller selection using the ANP MCDM. The second stage conducts a quantitative performance analysis of the top controller based on the high-weight values obtained from stage 1. The quantitative analysis consists of a comparison of topology discovery time, delay, throughput and CPU utilization with controller obtained through Analytical Hierarchy Process (AHP) by emulating the real-world internet and Brite topologies in Mininet. The ANP takes the feedback from the other cluster elements and dependency between them. The AHP has no mechanism for feedback and dependency between components [23]. We have used ANP which covers the feedback and dependency between components in the same and different clusters. The performance of the controller shall be validated in the real-world Internet and Brite topologies. In our previous work [24], we compared the performance of the POX and RYU controllers in various topologies. These topologies included Single, Linear, Tree, Dumbbell, and data center networks (DCNs). Herein, the approach is extended by first computing the optimum controller with respect to its supporting features using ANP and then conducting a performance analysis of the controller.

The rest of the paper is organized as follows: Section 2 describes work related to the selection of an SDN controller from a set of controllers. Section 3 presents a motivation for the feature-based controller selection. The proposed approach and contribution of the study is discussed in Section 4. Our proposed model for SDN controller selection, the analytical network process (ANP) approach, and the feature-based results are presented in Section 5. In Section 6, performance metrics, experimental setup and design are being discussed. In Section 7, the performance of two feature-based optimum controllers computed using our proposed and AHP approach is evaluated in real-Internet and Brite topologies. Finally, the paper is concluded in Section 8 based on the findings of the results.

## Literature review

In the literature, different approaches have been used for SDN controller selection. These approaches can be broadly classified into three categories. The first category involves comparing controllers based on their features, the second compares controllers based on their performance, and the third is a hybrid approach. The hybrid approach selects an optimum controller by combining the results of a feature and performance-based comparison. These approaches are discussed below.

The research studies presented in [7], [15–16] and [19–20] simply compare the performance of SDN controllers. The performance-based approaches only consider the performance merely neglecting the features of SDN controllers. Secondly, these approaches consider the performance in a general topology using Mininet or by creating virtual switches and hosts in Cbench. Therefore, the realistic scenario of real-world internet is not considered in their experiments.

The studies conducted in [25–28] considered features of controllers and provide a comparison of these controllers with respect to the supporting features that they offer. Examples of supporting features include the following: platform, REST API, clustering, and OpenFlow support. The goal of all approaches was to select an optimum SDN controller. However, approaches solely based on the feature set neglected the performance of SDN controllers. Another drawback of these approaches was that they only provide a theoretical analysis of the feature set provided by the controllers. Therefore, a comparative evaluation of these controllers can’t be made. The selection of an optimum controller considering its features presents several challenges. If we base our selection solely on the feature table of the controller, this will lead to cognitive overload. In this scenario, optimal decisions cannot be made owing to a limitation of the human capacity for information processing, which is commonly known as the 7 ± 2 problems, or Miler’s law [29].

A comparative study of four SDN controllers based on a hybrid approach was presented in [30]. The authors selected two controllers based on a heuristic decision from the controller feature table. They listed nine features supported by these controllers and selected two controllers based on a review of the features table. Then, they evaluated the performance of these controllers using Cbench by running it in throughput and latency mode. Their study didn’t provide the explicit ranking of these controllers as they have simply analyzed the feature table. There, a precise selection is not possible with their approach. Secondly, they have not considered the performance comparison in real-world Internet topologies. The study presented in [31] compared six SDN controllers, OPENDAYLIGHT (ODL), NOX, BEACON, MAESTRO, RYU, and LIBFLUIED RAW [32], to increase the number of threads and switches in both throughput and latency modes. A similar performance comparison study examined four controllers: RYU, Open Network Operating system (ONOS), ODL, and FLOODLIGHT. This study can be found in [33], and the authors used Cbench to measure the throughput and latency of each.

In [34], a comparative study of five SDN controllers, RYU, TREMA [35], FLOODLIGHT, ODL, and ONOS, was performed for aerial networks using qualitative and quantitative analysis. First, a qualitative study of these controllers was made with respect to two features i.e. clustering support and state handling mechanism. The state management information for five controllers was tabulated for checking how each controller gather, store the network state information and the status of this information in case a switch or the controller fails itself. i.e. whether the controller will reload this information from the previously saved state, or it will relearn the network status. Similarly, the information about the clustering mechanism for each controller was tabulated to check if these controllers support clustering and how different controllers share the information of the cluster they are managing. In their study, the top two controllers were selected based on the two feature that fulfilled the requirements of the aerial networks. A performance evaluation was conducted through an experimental scenario emulated in Mininet. However, their controller selection process was based on a heuristic decision and could lead to cognitive overload if the number of controllers and features scaled.

Multi-criteria decision-making (MCDM) is a mathematical decision-making technique where selection among several alternatives is made based on a set of criteria [36]. It has been widely used in various fields, such as in software development for selection of strategy [37], for managing natural resource [38], for network selection in heterogeneous networks [39], and several others. Different approaches are used for the selection process depends on a set of criteria to get the desired objective, for example, AHP, ANP, and several others. The selection of SDN controllers using an MCDM method such as AHP was proposed in [40]. The study considered ten controllers and ten features to select the controller based on their features. However, they did not consider any quantitative comparison of these controllers, and their paper does not provide any details on the approach they used.

A hybrid approach for controller selection based on AHP was described in [41]. In that study, the top three controllers were considered for performance test using Cbench; however, they didn’t evaluate the performance in real-Internet topologies. The authors did not provide the mathematical details of their methodology. In AHP, the feedback of the alternatives was not considered. Therefore, this feedback property is ignored in AHP, and it only focuses on the criteria for selection. Another drawback of AHP is that the criteria are treated independently, so a precise selection cannot be made. In [42], the ANP has been used for modeling risk factors in megaprojects using risk index. Similarly, Shah Nazir et al [43] applied it for selection of software component using the quality as a criteria. The ANP has been used for wireless sensors used in the selection of an optimum cluster head [44], we deduce that ANP approach can also be employed to analyze the systems with complex behavior and structure. The complexity of the systems has increased the dependency among them; therefore, the study of the interdependent systems is a burning matter in the network systems [45]. The ANP is a long-established tool in the decision-making process depends on several criteria.

## Motivations

The software-defined networking (SDN) is composed of data, control and management planes. However, the control plane plays the main role because it manages the data plane which is the actual network topology. Therefore, SDN controllers and their features are important for the performance of SDN. Each controller has several features. In this section, ten features are discussed which influence the performance of the SDN.

1. OpenFlow:—OpenFlow protocol (also known as the southbound API) manages the flow request messages sent by the data plane to the control plane and vice versa. The data plane is the actual network topology or the underlying network, and the control plane consists of the SDN controller. In response to the flow requests (PACKET_IN messages), the flow response (PACKET_OUT messages) are sent by the controller. Therefore, communication between these two planes is managed by the OpenFlow [1]. The research work presented in [46] has described that the request and response messages influence the delay of the controller. Therefore, they have proposed an efficient clustering approach for minimizing the overall end to end delay of the SDN by placing multiple controllers in the clusters with many switches. However, the OpenFlow version each controller supports is different e.g. The higher versions, i.e. v1.3 supports load balancing which helps in improving the performance during the generation of high traffic load.
2. GUI:—The GUI helps in viewing statistics of the underlying topology, configuration of the OpenFlow switch entries and applications management. The GUI is one of the key features in the selection of the SDN controller while making the qualitative and quantitative analysis of the controllers as illustrated in [30]. The SDN controllers have a command line interface (CLI) and GUI support. The GUI of the controller helps in viewing statistics such as the number of hosts and switches, OpenFlow entries, OpenFlow tables and the making of the SDN topologies [47]. The statistics are viewed in a user-friendly format which is easy to analyze. Similarly, flows can be pushed to the OpenFlow switches via this interface. However, the GUI feature influences the performance because GUI execution is slower than CLI. In the SDN controller scenario, there are two types of GUI support in each controller. One is Python-based and other is java supported web-based. The python coded controllers have less support for multithreading and memory access management; therefore, their execution speed is slower than the controllers having Java-based support. Therefore, the controller’s performance is influenced by the GUI support of the controller.
3. Northbound REST API:—The communication between the controller and the applications in the management plane takes place through the REST API also known as the northbound interface. Similarly, the operational statistics about the OpenFlow switches and the topology are gathered via this API. The controller acts as a bridge between the data plane and management planes using the REST API. Therefore, this feature plays an important role while the selection of the SDN controller because of its direct communication with the controller. The fast response of the API will result in a reduction of delay and improvement of throughput. Therefore, REST API plays a key role in the SDN performance and it is considered by [40, 41] for the SDN controller selection.
4. Clustering:—The innate support for clustering helps in scalability, reliability and improving the performance of the control plane. Controllers with support of clustering resulted in improved performance with respect to delay [48]. Similarly, the delay reduction with increasing the number of OpenFlow switches and during the high traffic loads was also observed with clustering.
5. Quantum API:—The users take advantage of the cloud services by invoking it remotely via the Internet. Therefore, competition has been found between the cloud service providers (CSPs) and network service providers (NSPs). A new economic model has been proposed in [49] which distinguishes the contest between the CSPs and NSPs. Similarly, the authors in [50] proposed an approach for provisioning the end to end performance with a cloud service composition model. The support of quantum API enables the SDN controller to leverage cloud computing. The controllers having built-in support for this API can leverage the cloud for high-performance computing and OpenStack networking using the Quantum API. Thus, controllers having quantum support has capabilities for parallel processing and fast memory access. As a result, the performance improves with an increase in the scalability of the SDN, i.e., with an increase in the number of OpenFlow switches. This feature was included in the research studies [40] for the SDN controller selection.
6. Synchronization:—This shows how efficiently the controller responds and stores the information for the OpenFlow switches in the data plane. This influences the topology discovery time which is an important metric in measuring the performance of the SDN [34]. The controllers have less topology discovery time improves the performance of the SDN.
7. Productivity:—is related to the ease of applications development and is related to the programming language in which the controller is coded. Although the application development is easy with python coded controllers however the lack of platform support and multi-threading makes them slow. Therefore, productivity has an inverse relation with the performance of the controller [31]. The python coded are more productive due to their ease of application development however java supported being less productive have high performance. This is due to the high-performance capability of java coded controllers such as multithreading, cross-platform support, fast memory access and inter-process communication (IPC).
8. Partnership support:—Several multinational and national organizations support different controllers. Therefore, IT organizations not only look for the technical considerations while selecting a controller but also on some key aspects such as the financial resources and the technical strengths associated with the development of the controller. An organization may not want to affect their SDN based solutions from a vendor who is not able to adapt itself according to the changing needs of the SDN market. Therefore, this feature has a vital role in controller selection, and it is considered by the research studies [31] while selecting the controller.
9. Platform support:—The platform support shows the compatibility of a controller to run across different operating systems such as Windows, Linux or Mac. Running through different platforms makes the controller able for multithreading, fast memory access, and flexible memory management which influence the performance of a controller. Running across different platforms also makes the clustering more efficient, because controllers can make a cluster through different platforms. Therefore, the delay reduces during the normal and high traffic load and the QoS improves [48].
10. Modularity:—The capability of making the main program into subroutines is known as modularity. The modularity support makes a controller more viable when dealing with large scale systems. The sub-modules can run in parallel resulting in faster execution and less response time. Especially it will help in improving the performance when the scalability increases. Therefore, this feature was considered in the literature [31] for the SDN controller selection.

## Proposed approach

**Fig 1:**
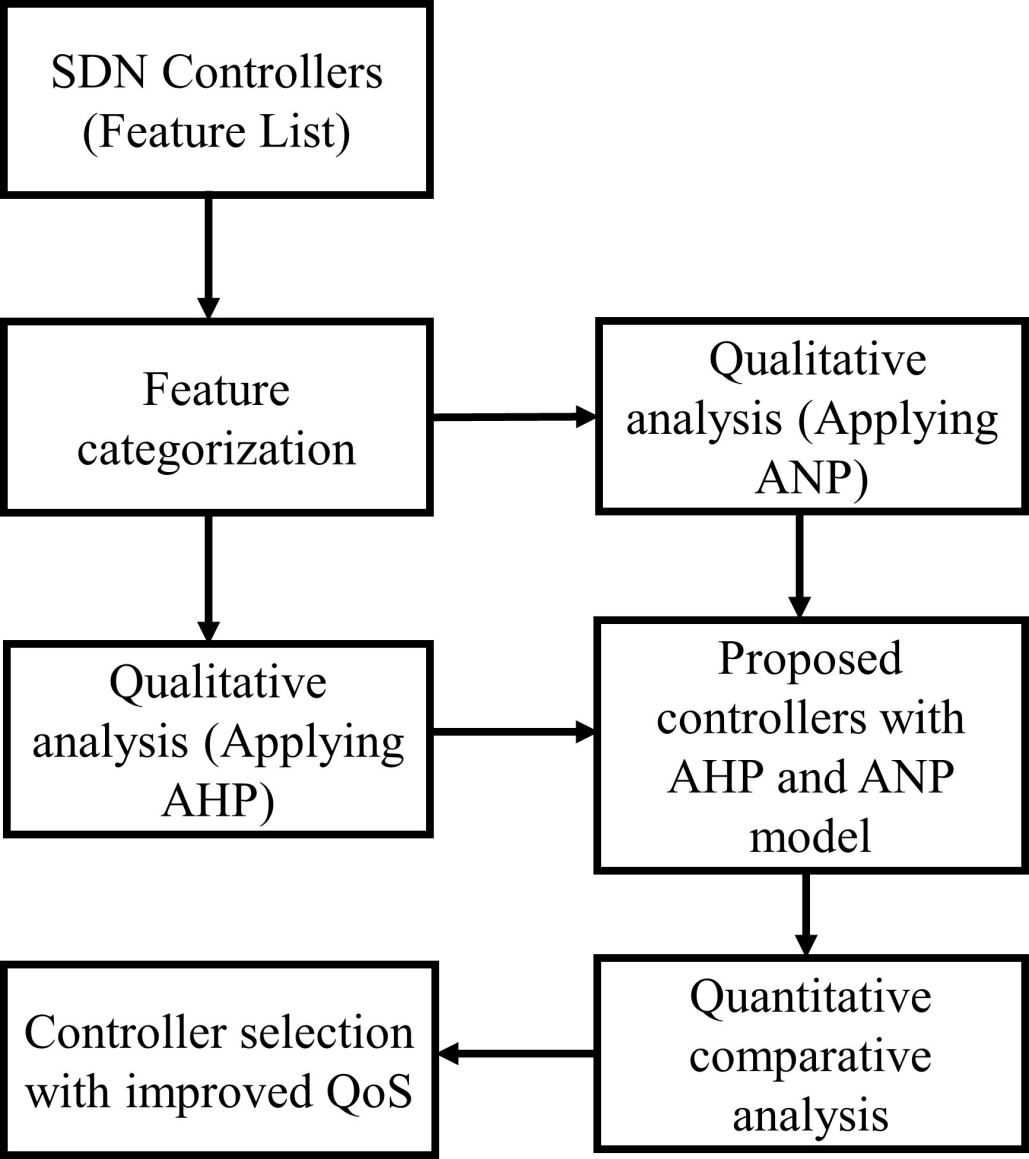
The proposed approach for selection of an optimum SDN controller.

The proposed approach for controller selection is based on the qualitative and quantitative analysis of the SDN controllers. A bird’s-eye view of the proposed method is illustrated in Fig 1. First, ANP is applied for the qualitative feature-based comparison of the SDN controllers. The ANP sorts the controllers with the provided feature set controllers by calculating weights for each controller. Further, the quantitative analysis of the high-weight controller is performed through several simulations in Mininet. The procedure to choose the optimum SDN controller is described below:

1. The SDN controllers are listed along with their features.
2. Then, features are categorized according to their support level in the SDN controller. Feature categorization is discussed in Section 5.2.
3. ANP is to be applied to rank the controllers i.e. from high weight to low weight values.
4. Then, the performance of the high-weight controller obtained through ANP was will be evaluated through several simulations.
5. Further, the performance comparison is to be done with the high-weight controller computed with AHP based approach for SDN controller selection.
6. The QoS parameters of the proposed controller with ANP and AHP based SDN controllers will be evaluated. Finally, the controller with optimum features and performance is selected.

The paper contributes to the controller selection problem while comparing SDN controllers by utilizing the features of the SDN controller using ANP. Secondly, the performance comparison of the controller selected with ANP and AHP was done for real-world and Brite topologies. The real-world and Brite generated topologies were converted to Mininet, and an experimental scenario was designed for topology discovery and delay evaluation. Furthermore, a delay calculation module was added for performance evaluation. Moreover, throughput and delay were evaluated with traffic generation scenario. Finally, the CPU utilization was reported for the controllers during the experiment of high traffic load evaluation.

## Analytical network process

The ANP was proposed by saaty [51]. It can be applied to the quantitative and qualitative data about a network. It can evaluate the feedback and dependency relationship between the criteria and alternatives. The ANP process for SDN controller selection is described in this subsection. The general procedure [52] for applying the ANP is given below.

**Fig 2:**
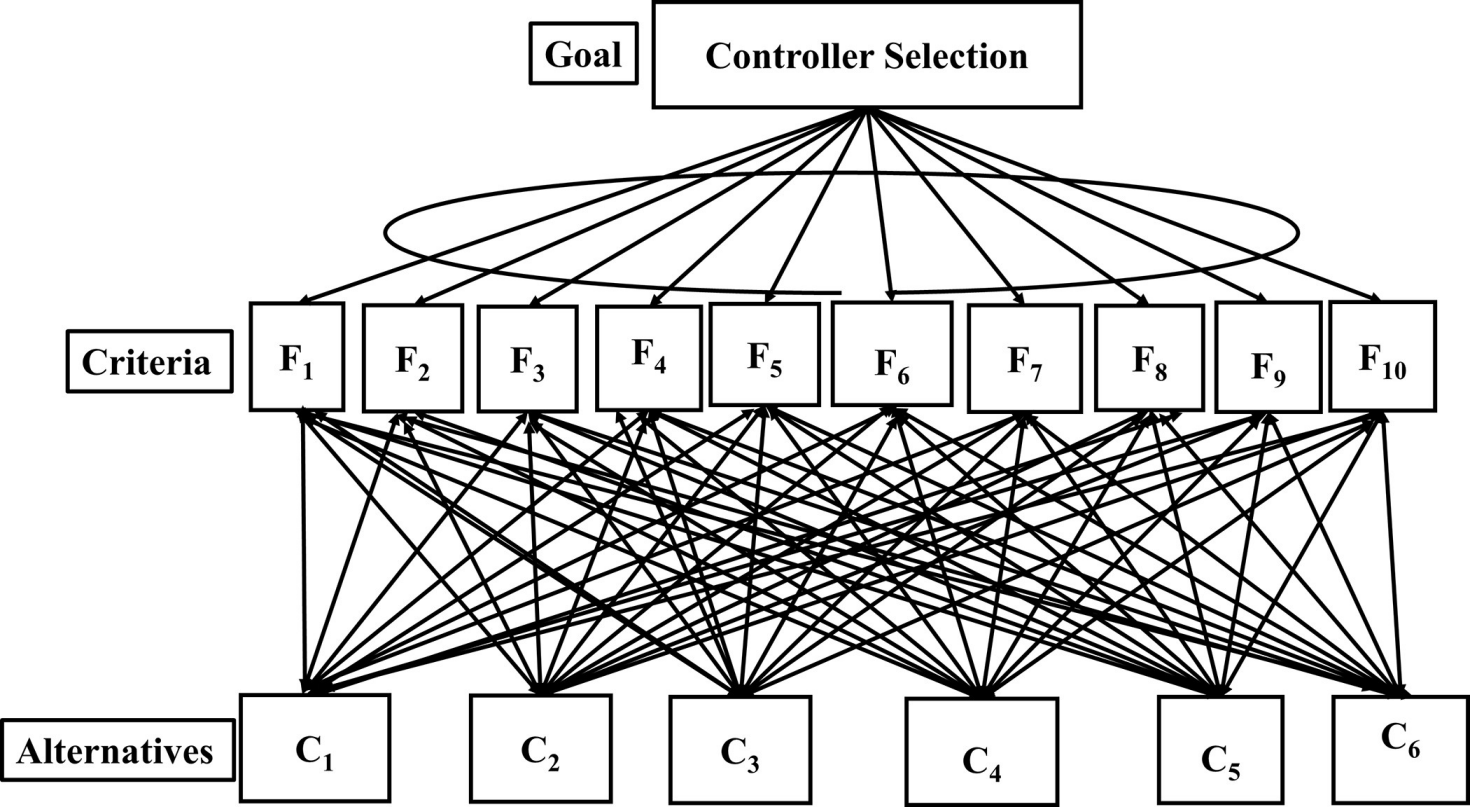
ANP Model for SDN controller selection.

1. The goal is set by formulating the problem, and then the criteria or sub-criteria needed to achieve the goal are identified. The goal is to rank the controllers according to their optimum features. Criteria are the feature list *(F)* of controller and alternatives are SDN controllers *(C)*. The selection of an alternative depends upon the criteria or its features. Table 1 and Table 2 describes the parameters of criteria and alternatives. The alternatives are ranked and assigned a weight based on their importance concerning the goal. The ANP model for controller selection is shown in Fig 2.
2. A qualitative scale is created for the criteria and alternatives that show the relative importance of each, and a quantitative range is defined for the qualitative scale described by saaty in [51], as shown in Table 3.
3. A pairwise comparison matrix is created where the rows *i* and columns *j* of the matrix have a value corresponding to *(i*, *j)*, which is derived from the scale table. This table shows the relative importance of the criterion over another. A value of *(i*, *j)* represents the relative importance of the criteria in the *i*^*th*^ row from the *j*^*th*^ column. The index *(j*, *i)* shows the importance of the criterion in the *j*^*th*^ column from the criterion in the *i*^*th*^ row. These values show the relative importance of the criteria or alternative to the goal. A value of 1 represents that both criteria are equally crucial whereas a value of 9 represents the extreme importance of one criterion over another.
4. The eigenvector, also known as the priority vector, shows the ranking of criteria or sub-criteria. It is computed by normalizing the columns of the comparison matrix and then taking the row averages.
5. The next step after calculating the eigenvector is to calculate the two most important parameters. These parameters are the consistency index *(CI)* and consistency ratio *(CR)*, proposed by satty in [53]. These parameters determine the reliability of the judgments, i.e., whether the results of the pairwise comparison matrix are consistent or not. Suppose we state that API is more significant than clustering support and clustering support is more critical than the GUI of a controller. Then, saying that GUI is more important than API would lead to an inconsistent judgment. Thus, to avoid such inconsistencies while making judgments, these two mentioned parameters are calculated for each pairwise comparison matrix. A *CR* of 0.1 or less implies that judgments are consistent. Otherwise, the pairwise comparisons are untrustworthy, and the process must be repeated.
6. The priority vectors obtained from the pairwise comparison matrix for both alternatives and criteria form an unweighted super-matrix, which will be converted to a weighted super-matrix by making the sum of each column equal to one.
7. The final output will be the limit super-matrix, that is to be obtained from the weighted super-matrix by taking its power until the matrix converges. Then, each block of the limit matrix is normalized to obtain the final weightage or priorities of all nodes (criteria and alternatives) under consideration. The high-weight value represents the controller with optimum features considered during the selection process.

**Table 1:** Parameters for criteria.

Serial#	Name	Terminology	Description
1	OpenFlow support	*F*_*1*_	OpenFlow 1.0–1.5
2	Graphical user interface	*F*_*2*_	Python based, Web based.
3	Northbound API support	*F*_*3*_	REST API.
4	Clustering support	*F*_*4*_	To avoid single point of failure (reliability)
5	Openstack networking	*F*_*5*_	Enabling different network technologies via Quantum API.
6	Synchronization	*F*_*6*_	State synchronization of the network.
7	Productivity	*F*_*7*_	For developing software.
8	Partnership support	*F*_*8*_	Cisco, IBM, Intel, Linux Foundation and Juniper.
9	Platform support	*F*_*9*_	Windows, Linux, Mac.
10	Modularity support	*F*_*10*_	The extent of dividing the code in submodules.

**Table 2:** Parameters for alternatives.

Serial#	Name	Terminology
1	FLOODLIGHT	*C*_*1*_
2	ODL	*C*_*2*_
3	ONOS	*C*_*3*_
4	POX	*C*_*4*_
5	RYU	*C*_*5*_
6	TREMA	*C*_*6*_

**Table 3:** Scale of importance.

Scale	Description
1	Equally important
2	Equally to moderately more important
3	Moderately more important
4	Moderately to significantly more important
5	Significantly more important
6	Significantly to remarkably more important
7	Remarkably more important
8	Remarkably to excessively more important
9	Excessively more important

### Problem formulation

The performance of SDN depends on the controller directly. Therefore, the selection of an optimum SDN controller will ensure effective network utilization, thus improving the quality of service (QoS). Each controller has several features such as OpenFlow, Platform support, South bound, and North bound Interface. Similarly, each controller has different platform support, such as POX supports Linux, Windows, and Mac, while TREMA only supports Linux. Likewise, each controller has a different OpenFlow version support (e.g., 1.0, 1.1, 1.2, etc.). The controller plays a prominent role in SDN; therefore, it should be selected carefully.

**Fig 3:**
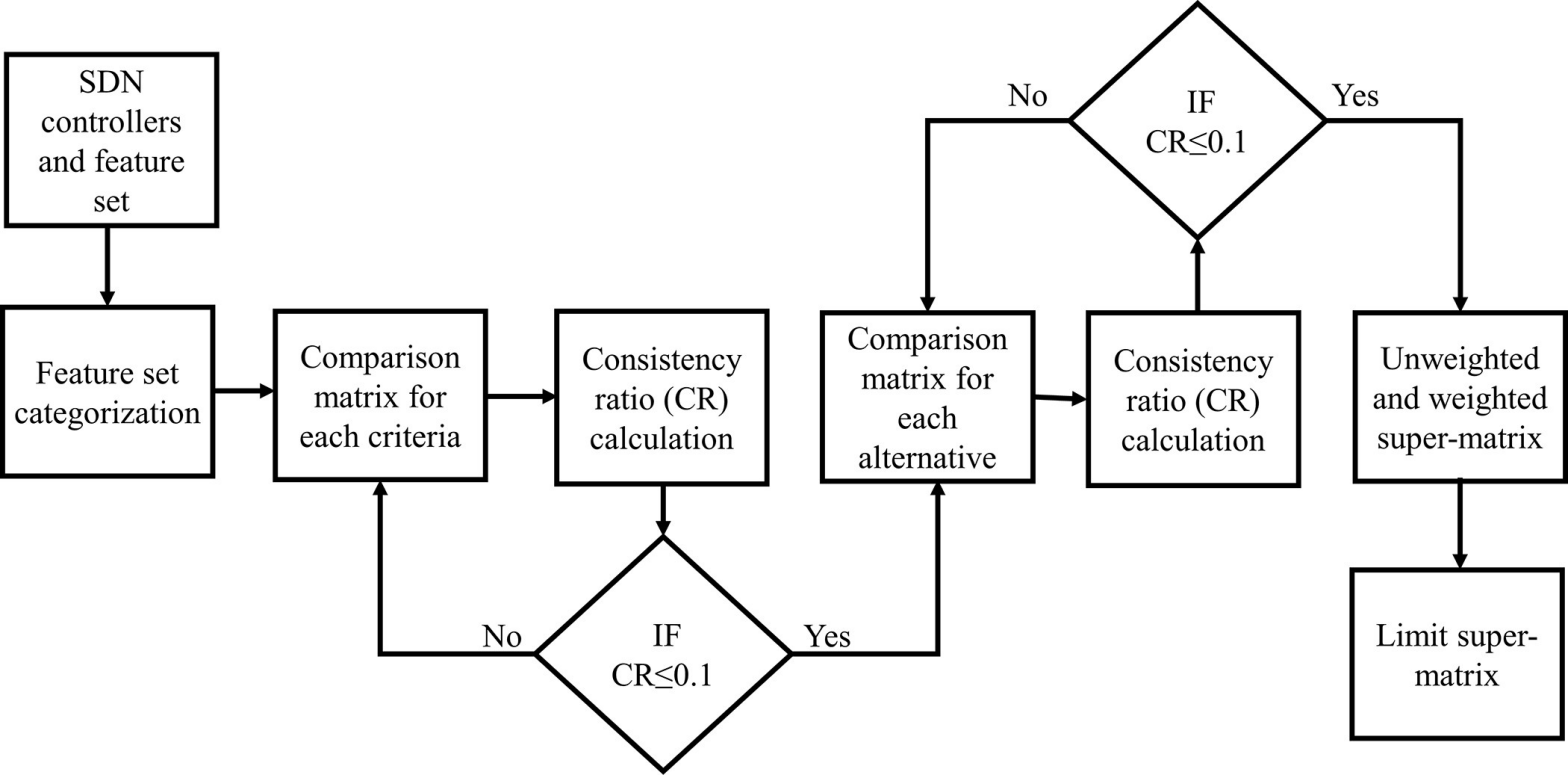
Step by step procedure for applying ANP in SDN controller selection process.

As each controller has several features; therefore, computing an optimum controller is considered as an MCDM problem. ANP is widely used in multi-criteria decision-making problems where the feedback of alternatives and dependency among criteria elements is considered. Before deployment of controller, the ANP algorithm will select the optimum controller among a set of controllers. Fig 3 shown the whole procedure for applying ANP in computing an optimum SDN controller based on their features, which will be evaluated experimentally in next section concerning the QoS metrics. Therefore, the controller which has an optimum feature set and improved QoS will be selected. The procedure for ranking these controllers according to their optimum feature set is as follows:

### Application of ANP in SDN controller selection

The ANP MCDM problem is formulated by first setting the goal or objective, then by defining the parameters for criteria or sub criteria, and finally the alternatives under evaluation are set, as shown in Fig 2. In this study, our objective is to select the optimum SDN controller with respect to the ten features as shown in Table 1 and Table 2. The criteria and alternatives are represented by Eqs (1) and (2). The available features offered by the different SDN controllers are denoted by *F*, and the alternatives from Eq (2) are denoted by *C*. A network model is made representing the criteria and alternatives as well as the relationship among them. The network model compares each alternative with respect to each criteria and vice versa.

$$F=(F_{1},F_{2},F_{3},\ldots ,F_{N})$$ (1)

$$C=(C_{1},C_{2},C_{3},\ldots ,C_{N})$$ (2)

The 10 essential features that should be considered in SDN controller selection as a criteria were described in [54]. Therefore, we assume that all these features are required for the controller selection process. However, as controllers are always evolving, we considered the latest information about these features from the controller documentation and research in [40, 41]. Herein, these important features are utilized and considered in the optimum controller selection process using ANP. Therefore, the importance of a feature in every controller is identified by categorizing these features.

Features supported by a controller are divided into two types: (1) Ordinal and (2) Regular categorical. Ordinal features are those that have an inherent ordering while regular categorical features don’t have an inherent ordering to them. Categorization of the feature set gives a clear insight into the support level of that feature in each controller. For example, *C*_*4*_ and *C*_*6*_ support only OpenFlow v1.0 therefore with respect to this feature *(F*_*1*_*)* they are kept in the low category as shown in Table 4. *C*_*1*_ support is medium, *C*_*2*_ and *C*_*3*_ support v1.0,1.1,1.3, therefore, they are kept in the high category, *C*_*5*_ supports higher versions of OpenFlow i.e. 1.5, therefore, it is placed in very high category for this feature. *F*_*2*_ represents the GUI feature of a controller. *C*_*1*_ has support for java supported web-interface and it executes faster because of the basic graphical functions for application and data plane management. Therefore, it is placed in the ‘Very High’ category concerning *F*_*2*_ because it leverages the Java multithreading. Similarly, *C*_*2*_ and *C*_*3*_ has support for Java-based interface and supports QoS parameters settings for the data plane devices. They have more functions for applications management and topology configuration which makes their GUI comparatively slow than *C*_*1*_. Therefore a ‘High’ category is assigned to them. The *C*_*4*_ supports python-based interface, and its execution speed is faster than *C*_*5*_ and *C*_*6*_ because of its preliminary functions; therefore, it is placed in the ‘Medium’ category. *C*_*5*_ and *C*_*6*_ support only python-based interface; however, they run slowly due to more functions for handling the data plane and management plane and lack of multithreading.

**Table 4:** Features for SDN controller selection.

Alternatives	Criteria/Features									
	*F*_*1*_	*F*_*2*_	*F*_*3*_	*F*_*4*_	*F*_*5*_	*F*_*6*_	*F*_*7*_	*F*_*8*_	*F*_*9*_	*F*_*10*_
*C*_*1*_	Medium	Very High	Yes	Yes	No	Medium	Medium	Low	Low	Medium
*C*_*2*_	High	High	Yes	Yes	Yes	Medium	Medium	Very High	High	High
*C*_*3*_	High	High	Yes	Yes	Yes	Low	Medium	High	High	High
*C*_*4*_	Low	Medium	No	No	No	Low	High	Low	High	Low
*C*_*5*_	Very High	Low	No	Yes	No	High	High	Medium	Low	Medium
*C*_*6*_	Low	Low	No	Yes	No	Low	High	Low	Low	Medium

*F*_*3*_, *F*_*4*_, and *F*_*5*_ are the regular categorical features, i.e. a particular feature which can't be further divided into further levels. For example, a controller may or may not have the support for REST API, open stack networking and clustering. Therefore, these features (*F*_*3*_, *F*_*4*_, and *F*_*5*_) doesn’t have an inherent ordering. These features are represented with a 'Yes' or 'No' in Table 4. Like *C*_*4*_, *C*_*5*_ and *C*_*6*_ don’t have built-in support for REST API; therefore a ‘No’ is placed for them in Table 4 corresponding *F*_*3*_ field. Similarly, *C*_*1*_, *C*_*2*_, and *C*_*3*_ have built-in support for the REST API; therefore a 'Yes' is written in the *F*_*3*_ column. *F*_*4*_ shows the Quantum API feature. *C*_*1*_, *C*_*2*_, *C*_*3*_, *C*_*5*_, and *C*_*6*_ have an innate Quantum API support, therefore 'Yes' is shown in the column *F*_*4*_ for them. *C*_*4*_ doesn’t have support for the Quantum API; therefore ‘No’ is written in *F*_*4*_ corresponding to this controller. F_5_ denotes the clustering feature. The controllers *C*_*1*_, *C*_*4*_, *C*_*5*_, and *C*_*6*_, does not have built-in support for clustering; therefore a ‘No’ is placed in Table 4 for them in *F*_*5*_ field. In contrast, *C*_*2*_ and *C*_*3*_ support clustering; therefore, they are represented with 'Yes'.

*F*_*6*_ represents the synchronization feature which influences the topology discovery and response from the data plane. *C*_*1*_, *C*_*5*,_ and *C*_*6*_ have a medium level, i.e. their interaction is relatively slower. The *C*_*2*_ and *C*_*3*_ have high level because of their fast discovery of the underlying topology while *C*_*4*_ has a low level because of the slowest interaction between the data and control plane. *F*_*7*_ denotes the productivity level of a controller. Productivity is related to the ease of applications development and is associated with the programming language in which the controller is coded. Although the application development is easy with python coded controllers; however, the lack of platform support, memory management and multi-threading makes them slow. *C*_*1*_, *C*_*2*,_ and *C*_*3*_ have a medium productivity level while *C*_*4*_, *C*_*5*,_ and *C*_*6*_ have a high level of productivity.

*F*_*8*_ denotes the support from different vendors *C*_*2*_ has support from Cisco, NEC, IBM, and Linux foundation whose membership spans to 40 companies; therefore, it is placed in the ‘Very high’ category. *C*_*3*_ is supported by Sk telecom (South Korean telecommunication), Cisco and NEC; therefore, it is placed in the ‘high’ category. *C*_*1*_, *C*_*4*,_ and *C*_*6*_ are supported by Big Switch Networks, Nicira, and NEC, i.e., therefore, they are placed in the ‘Low’ category with respect to their support level. Although it is not directly related to performance, However, good support from the vendor eventually results in improving the performance as discussed in section 3. *F*_*9*_ represents platform support. *C*_*2*_, *C*_*3*,_ and *C*_*4*_ have support in three platforms, i.e. Linux, Mac, and windows, therefore are placed in the high category. However, *C*_*1*_, *C*_*5*,_ and *C*_*6*_ are supported in only one platform i.e. Linux, therefore a low category is assigned to them. As discussed in section 3 the cross-platform support enables multithreading and clustering support resulting in improved QoS. *F*_*10*_ denotes modularity support. *C*_*1*_, *C*_*5*,_ and *C*_*6*_ have a medium level for modularity while *C*_*2*_ and *C*_*3*_ have a high level because of *C*_*2*_ and *C*_*3*_ controllers can make a call to the submodules from the main function resulting in parallel processing and consequently increase the performance. Feature categorization is done as a preprocessing step before making the comparison matrix.

#### Pairwise comparison matrix for criteria and alternatives

The pairwise comparison matrix is made according to the 9-point scale proposed by saaty [51]. It shows the relative importance of different components (criteria or alternatives) regarding an element. The same matrix is also employed to extrapolate the effect of the components on the objective using the 9-point scale as shown in Table 3. The values in the matrix are assigned to the criteria as well as alternatives represents personal judgments. For example, how much *C*_*1*_ is important from *C*_*2*_, *C*_*3*_, *C*_*4*,_ and *C*_*5*_ with respect to *F*_*1*_ component is assigned a value from the scale table. The value of *a*_(*i*,*j*)_ represents the relative significance of a component corresponding to the *i*^*th*^ row and row *j*^*th*^ column. The value of *a*_(*i*,*j*)_ = 1 in the pairwise comparison matrix shows the equal importance of the component corresponding to *i*^*th*^ row and *j*^*th*^ column. The diagonal components correspond to the comparison of the same components; therefore, their values are 1. The values below the diagonal are the reciprocal of the values above the diagonal. The value of, *a*_(5,1)_ = 6 in the matrix (4) shows that component in the 5^th^ row is significant to remarkably more important than the component in the 1^st^ column. The value of $a_{\left(1,5\right)}=\frac{1}{a_{\left(5,1\right)}}=\frac{1}{6}$ is the reciprocal of *a*_(5,1)_, denotes that component in the 1^st^ row is significant to remarkably less important than component in the 5^th^ column. The values are incorporated prudently for all the components in the pairwise comparison matrix.

#### Pairwise comparison of alternatives with respect to criteria components

Alternatives are pairwise compared for each criteria component. The general form of the pairwise comparison matrix is denoted in the matrix (3). The rows and columns of the matrix are represented as *M*_*1*_ to *M*_*n*_ and *N*_*1*_ to *N*_*n*_. First, the alternatives are pairwise compared with respect to *F*_*1*_ criterion. The values have been incorporated in (3) based using the 9-point scale defined in Table 3. The resultant values are shown in a matrix (4). The nonreciprocal and reciprocal values indicate the relative importance of the row and column components respectively. In matrix (4) the comparison of *C*_*1*_ is made with *C*_*2*_, *C*_*3*_, *C*_*4*_, *C*_*5*_, and *C*_*6*_ considering *F*_*1*_ criterion. *C*_*1*_ is of the same importance with itself therefore *a*_(1,1)_ *= 1*. Then *C*_*2*_ and *C*_*3*_ are moderately more important than *C*_*1*_. i.e. $\;a_{\left(1,2\right)}=a_{\left(1,3\right)}=\frac{1}{3}$. *C*_*1*_ moderately more important than *C*_*4*_ and *C*_*6*_, e.g. *a*_(1,6)_ *= 3* shows that alternative in this row *(C*_*1*_*)* is moderately more important than the alternative in the corresponding column *(C*_*6*_*)*. $a_{\left(1,5\right)}=\frac{1}{6}$ shows that *C*_*5*_ is significantly to remarkably more important than *C*_*1*_. Similarly, the values are filled for *C*_*2*_, *C*_*3*_, *C*_*4*_, *C*_*5*_, and *C*_*6*._

$$\left[\begin{array}{cccccc} & N_{1} & N_{2} & N_{3} & \cdots & N_{n}\\ M_{1} & 1 & a_{\left(1,2\right)} & a_{\left(1,3\right)} & \cdots & a_{\left(1,n\right)}\\ M_{2} & \frac{1}{a_{\left(1,2\right)}} & 1 & a_{\left(2,3\right)} & \cdots & a_{\left(2,n\right)}\\ M_{3} & \frac{1}{a_{\left(1,3\right)}} & \frac{1}{a_{\left(2,3\right)}} & 1 & \cdots & a_{\left(3,n\right)}\\ \vdots & \vdots & \vdots & \vdots & \ddots & \vdots \\ M_{n} & \frac{1}{a_{\left(1,n\right)}} & \frac{1}{a_{\left(2,n\right)}} & \frac{1}{a_{\left(3,n\right)}} & \cdots & 1 \end{array}\right]$$ (3)

matrix (4) is the resultant of all comparisons of the alternatives for *F*_*1*_ criterion. Each column in the matrix (4) is summed up and each value is divided by the sum of the total values of the column according to the matrix (5). The result is a normalized matrix illustrated in the matrix (6).

$$\left[\begin{array}{ccccccc} & C_{1} & C_{2} & C_{3} & C_{4} & C_{5} & C_{6}\\ C_{1} & 1 & \frac{1}{3} & \frac{1}{3} & 3 & \frac{1}{6} & 3\\ C_{2} & 3 & 1 & 1 & 6 & \frac{1}{3} & 6\\ C_{3} & 3 & 1 & 1 & 6 & \frac{1}{3} & 6\\ C_{4} & \frac{1}{3} & \frac{1}{6} & \frac{1}{6} & 1 & \frac{1}{9} & 1\\ C_{5} & 6 & 3 & 3 & 9 & 1 & 9\\ C_{6} & \frac{1}{3} & \frac{1}{6} & \frac{1}{6} & 1 & \frac{1}{9} & 1 \end{array}\right]$$ (4)

$$\left[\begin{array}{ccc} \frac{a_{\left(1,1\right)}}{\sum_{i=1}^{n}a_{\left(i,1\right)}} & \cdots & \frac{a_{\left(1,n\right)}}{\sum_{i=1}^{n}a_{\left(i,n\right)}}\\ \vdots & \ddots & \vdots \\ \frac{a_{\left(n,1\right)}}{\sum_{i=1}^{n}a_{\left(i,1\right)}} & \cdots & \frac{a_{\left(n,n\right)}}{\sum_{i=1}^{n}a_{\left(i,n\right)}} \end{array}\right]$$ (5)

$$\left[\begin{array}{ccccccc} & C_{1} & C_{2} & C_{3} & C_{4} & C_{5} & C_{6}\\ C_{1} & \frac{1}{13.67} & \frac{0.33}{5.67} & \frac{0.33}{5.67} & \frac{3}{26} & \frac{0.167}{2.06} & \frac{3}{26}\\ C_{2} & \frac{3}{13.67} & \frac{1.00}{5.67} & \frac{1.00}{5.67} & \frac{6}{26} & \frac{0.33}{2.06} & \frac{6}{26}\\ C_{3} & \frac{3}{13.67} & \frac{1.00}{5.67} & \frac{1.00}{5.67} & \frac{6}{26} & \frac{0.33}{2.06} & \frac{6}{26}\\ C_{4} & \frac{0.33}{13.67} & \frac{0.167}{5.76} & \frac{0.167}{5.76} & \frac{1}{26} & \frac{0.11}{2.06} & \frac{1}{26}\\ C_{5} & \frac{6.00}{13.67} & \frac{3}{5.76} & \frac{3}{5.76} & \frac{9}{26} & \frac{1}{2.06} & \frac{9}{26}\\ C_{6} & \frac{0.33}{13.67} & \frac{0.167}{5.76} & \frac{0.167}{5.76} & \frac{1}{26} & \frac{0.11}{2.06} & \frac{1}{26} \end{array}\right]$$ (6)

The Eigenvector *X* is obtained from this normalized matrix (6) according to Eq (7).

$$X_{i}=\frac{1}{n}\sum_{j=1}^{n}a_{\left(i,j\right)},\;\mathrm{where}\;i=1,2,3,\ldots ,n$$ (7)

$$\left[\begin{array}{cccccccc} & C_{1} & C_{2} & C_{3} & C_{4} & C_{5} & C_{6} & X_{1}\\ C_{1} & 0.073 & 0.059 & 0.059 & 0.115 & 0.081 & 0.115 & 0.084\\ C_{2} & 0.220 & 0.176 & 0.176 & 0.231 & 0.162 & 0.231 & 0.199\\ C_{3} & 0.220 & 0.176 & 0.176 & 0.231 & 0.162 & 0.231 & 0.199\\ C_{4} & 0.024 & 0.029 & 0.029 & 0.038 & 0.054 & 0.038 & 0.036\\ C_{5} & 0.439 & 0.529 & 0.529 & 0.346 & 0.486 & 0.346 & 0.446\\ C_{6} & 0.024 & 0.029 & 0.029 & 0.038 & 0.054 & 0.038 & 0.036 \end{array}\right]$$ (8)

The result from Eq (7) which is considered as the eigenvector *X*_*1*_ is represented in matrix (8). To verify whether the judgments made while making the pairwise matrix are consistent, the next step is to find the *CI* and *CR* values. However, before making the consistency analysis, the consistency measure *(CM)* vector is to be calculated.

**Fig 4:**
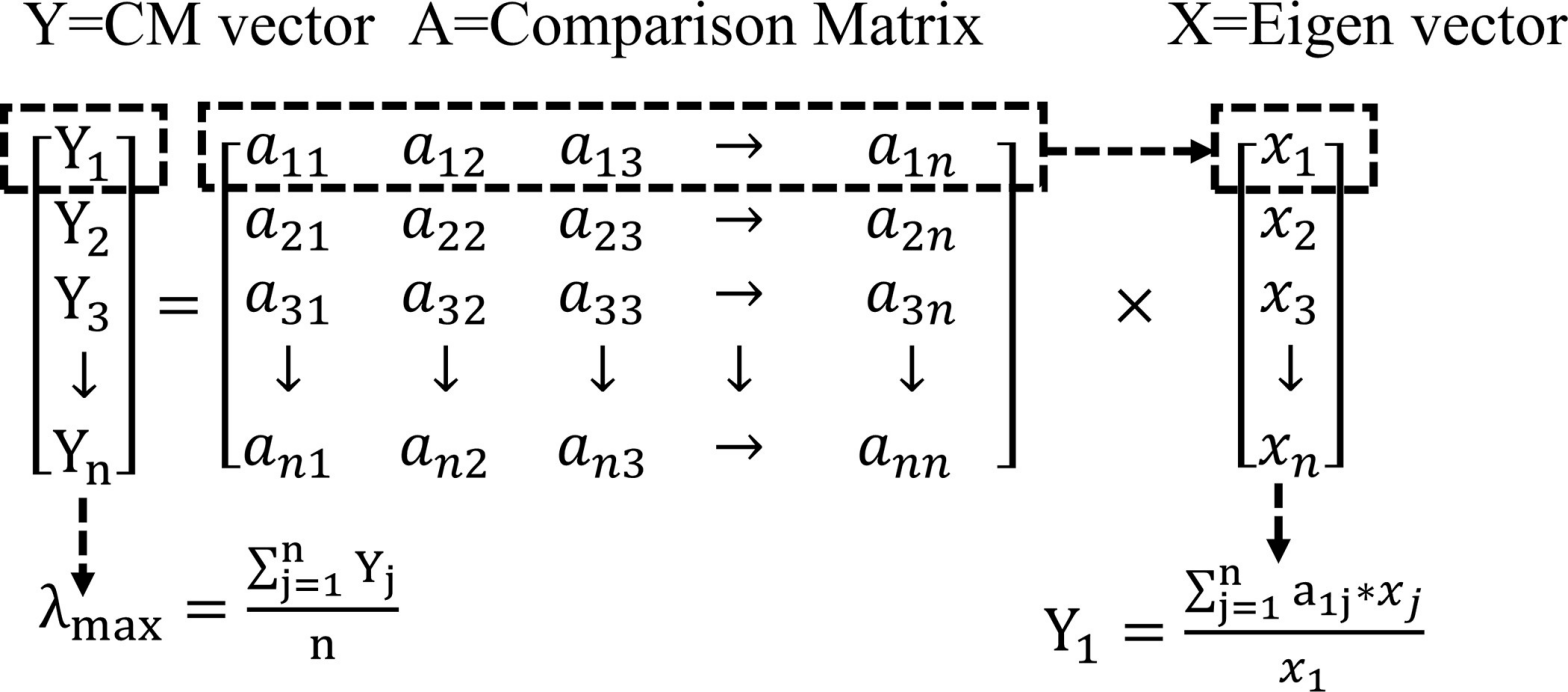
Calculation of consistency measure.

Consistency Measure: The *CM* vector is a prerequisite for calculation of *CI* and *CR*. The consistency measure is calculated according to Eq (9). The *M*_*j*_ denotes the row of the comparison matrix (4). The *X* and *x*_*i*_ represents the Eigenvector and the corresponding element of the Eigenvector as shown in the matrix (8). The *M*_*j*_ and *X* are multiplied and then divided by the component in the Eigenvector corresponding to *M*_*j*_. The procedure to find the *CM* is shown in Fig 4. The *CM* vector is averaged for computing *λ*_*max*_.

$$Y_{j}=\frac{M_{j}*X}{x_{i}},\;\mathrm{where}\;j=1,2,3,\ldots ,n$$ (9)

$$\lambda_{max}=\frac{1}{n}\sum_{j=1}^{n}Y_{j}$$ (10)

Consistency Index: The *CI* denotes the deviation or the inconsistency [51] of the pairwise comparison matrix for an element. The *CI* of the pairwise comparison matrix for *F*_*1*_ criterion is calculated using Eq (11) by putting the value of *λ*_*max*_. The value of *λ*_*max*_ = 6.07 and *n = 6* is put in Eq (11).

$$CI=\frac{\left(\lambda_{max}-n\right)}{\left(n-1\right)}$$ (11)

In Eq (11), n represents the criterion number for controller selection in the comparison matrix. Herein, six alternatives are considered; therefore, n is equal to 6. The resultant value for *CI = 0*.*01* according to Eq (11).

Consistency Ratio: The reliability of the pairwise comparison matrix is verified by calculating the *CR* value. The *CR* is calculated according to Eq (12). In Eq (12) the ratio index *(RI)* denotes the index ratio. The value of *RI = 1*.*24* is derived from Table 5, based on the order of the matrix. If the rank of the matrix is three (the actual number of alternatives being compared), then a value corresponding to three is selected for *RI*. In this case, the number of criteria under consideration is 6. Therefore, a value corresponding to 6 will be inserted from Table 5. The *CR* is derived by putting *CI* value from Eq (11) in Eq (12).

$$CR=\frac{CI}{RI}$$ (12)

The *CR* value is 0.09. A *CR* of 0.1 or less is accepted for the inconsistent judgments of the comparison matrix; otherwise, the inconsistency is considered to be high and pairwise judgments must be made again to satisfy the condition, i.e. *CR ≤ 0*.*1*. The *CR* for matrix (13) is 0.09 which is less than 0.1; therefore, the judgments are pairwise consistent in the comparison matrix. The alternatives are pairwise compared for remaining criteria, i.e. *F*_*2*_, *F*_*3*_, *F*_*4*_, *F*_*5*_, *F*_*6*_, *F*_*7*_, *F*_*8*_, *F*_*9*_ and *F*_*10*_ as shown in matrix (14)-(22). The *CI* and *CR* values are computed using the same process for each of these matrices. The *CR* value is shown in each matrix.

**Table 5:** Ratio index for different number of criteria.

No. of Criteria	1	2	3	4	5	6	7	8	9	10
**Ratio Index**	0.00	0.00	0.58	0.90	1.12	1.24	1.32	1.41	1.45	1.49

$$\left[\begin{array}{cccccccc} & C_{1} & C_{2} & C_{3} & C_{4} & C_{5} & C_{6} & X_{1}\\ C_{1} & 0.073 & 0.059 & 0.059 & 0.115 & 0.081 & 0.115 & 0.084\\ C_{2} & 0.220 & 0.176 & 0.176 & 0.231 & 0.162 & 0.231 & 0.199\\ C_{3} & 0.220 & 0.176 & 0.176 & 0.231 & 0.162 & 0.231 & 0.199\\ C_{4} & 0.024 & 0.029 & 0.029 & 0.038 & 0.054 & 0.038 & 0.036\\ C_{5} & 0.439 & 0.529 & 0.529 & 0.346 & 0.486 & 0.346 & 0.446\\ C_{6} & 0.024 & 0.029 & 0.029 & 0.038 & 0.054 & 0.038 & 0.036\\ CR & 0.09 \end{array}\right]$$ (13)

$$\left[\begin{array}{cccccccc} & C_{1} & C_{2} & C_{3} & C_{4} & C_{5} & C_{6} & X_{2}\\ C_{1} & 1 & 3 & 3 & 6 & 7 & 9 & 0.421\\ C_{2} & \frac{1}{3} & 1 & 1 & 4 & 5 & 9 & 0.207\\ C_{3} & \frac{1}{3} & 1 & 1 & 4 & 5 & 9 & 0.207\\ C_{4} & \frac{1}{6} & \frac{1}{4} & \frac{1}{4} & 1 & 3 & 9 & 0.091\\ C_{5} & \frac{1}{7} & \frac{1}{5} & \frac{1}{5} & \frac{1}{3} & 1 & 5 & 0.049\\ C_{6} & \frac{1}{9} & \frac{1}{9} & \frac{1}{9} & \frac{1}{9} & \frac{1}{5} & 1 & 0.020\\ CR & 0.09 \end{array}\right]$$ (14)

$$\left[\begin{array}{cccccccc} & C_{1} & C_{2} & C_{3} & C_{4} & C_{5} & C_{6} & X_{3}\\ C_{1} & 1 & 1 & 1 & 9 & 9 & 9 & 0.300\\ C_{2} & 1 & 1 & 1 & 9 & 9 & 9 & 0.300\\ C_{3} & 1 & 1 & 1 & 9 & 9 & 9 & 0.300\\ C_{4} & \frac{1}{9} & \frac{1}{9} & \frac{1}{9} & 1 & 1 & 1 & 0.333\\ C_{5} & \frac{1}{9} & \frac{1}{9} & \frac{1}{9} & 1 & 1 & 1 & 0.333\\ C_{6} & \frac{1}{9} & \frac{1}{9} & \frac{1}{9} & 1 & 1 & 1 & 0.333\\ CR & 0.00 \end{array}\right]$$ (15)

$$\left[\begin{array}{cccccccc} & C_{1} & C_{2} & C_{3} & C_{4} & C_{5} & C_{6} & X_{4}\\ C_{1} & 1 & 1 & 1 & 9 & 1 & 1 & 0.195\\ C_{2} & 1 & 1 & 1 & 9 & 1 & 1 & 0.195\\ C_{3} & 1 & 1 & 1 & 9 & 1 & 1 & 0.195\\ C_{4} & \frac{1}{9} & \frac{1}{9} & \frac{1}{9} & 1 & \frac{1}{9} & \frac{1}{9} & 0.021\\ C_{5} & 1 & 1 & 1 & 9 & 1 & 1 & 0.195\\ C_{6} & 1 & 1 & 1 & 9 & 1 & 1 & 0.195\\ CR & 0.00 \end{array}\right]$$ (16)

$$\left[\begin{array}{cccccccc} & C_{1} & C_{2} & C_{3} & C_{4} & C_{5} & C_{6} & X_{5}\\ C_{1} & 1 & 1 & \frac{1}{3} & 6 & \frac{1}{3} & 3 & 0.139\\ C_{2} & 1 & 1 & 3 & 6 & \frac{1}{3} & 3 & 0.190\\ C_{3} & 3 & \frac{1}{3} & 1 & 3 & \frac{1}{6} & 1 & 0.128\\ C_{4} & \frac{1}{6} & \frac{1}{6} & \frac{1}{3} & 1 & \frac{1}{9} & \frac{1}{3} & 0.030\\ C_{5} & 3 & 3 & 6 & 9 & 1 & 6 & 0.439\\ C_{6} & \frac{1}{3} & \frac{1}{3} & 1 & 3 & \frac{1}{6} & 1 & 0.071\\ CR & 0.09 \end{array}\right]$$ (17)

$$\left[\begin{array}{cccccccc} & C_{1} & C_{2} & C_{3} & C_{4} & C_{5} & C_{6} & X_{6}\\ C_{1} & 1 & \frac{1}{6} & \frac{1}{6} & 1 & 1 & 1 & 0.045\\ C_{2} & 6 & 1 & 1 & 9 & 9 & 9 & 0.409\\ C_{3} & 6 & 6 & 1 & 9 & 9 & 9 & 0.409\\ C_{4} & 1 & \frac{1}{9} & \frac{1}{9} & 1 & 1 & 1 & 0.045\\ C_{5} & 1 & \frac{1}{9} & \frac{1}{9} & 1 & 1 & 1 & 0.045\\ C_{6} & 1 & \frac{1}{9} & \frac{1}{9} & 1 & 1 & 1 & 0.045\\ CR & 0.00 \end{array}\right]$$ (18)

$$\left[\begin{array}{cccccccc} & C_{1} & C_{2} & C_{3} & C_{4} & C_{5} & C_{6} & X_{7}\\ C_{1} & 1 & 1 & 1 & \frac{1}{3} & \frac{1}{3} & \frac{1}{3} & 0.083\\ C_{2} & 1 & 1 & 1 & \frac{1}{3} & \frac{1}{3} & \frac{1}{3} & 0.083\\ C_{3} & 1 & 1 & 1 & \frac{1}{3} & \frac{1}{3} & \frac{1}{3} & 0.083\\ C_{4} & 3 & 3 & 3 & 1 & 1 & 1 & 0.250\\ C_{5} & 3 & 3 & 3 & 1 & 1 & 1 & 0.250\\ C_{6} & 3 & 3 & 3 & 1 & 1 & 1 & 0.250\\ CR & 0.00 \end{array}\right]$$ (19)

$$\left[\begin{array}{cccccccc} & C_{1} & C_{2} & C_{3} & C_{4} & C_{5} & C_{6} & X_{8}\\ C_{1} & 1 & \frac{1}{9} & \frac{1}{6} & 1 & \frac{1}{3} & 1 & 0.043\\ C_{2} & 9 & 1 & 3 & 9 & 6 & 9 & 0.507\\ C_{3} & 6 & \frac{1}{3} & 1 & 6 & 3 & 6 & 0.251\\ C_{4} & 1 & \frac{1}{9} & \frac{1}{6} & 1 & \frac{1}{3} & 1 & 0.043\\ C_{5} & 3 & \frac{1}{6} & \frac{1}{3} & 3 & 1 & 3 & 0.109\\ C_{6} & 1 & \frac{1}{9} & \frac{1}{6} & 1 & \frac{1}{3} & 1 & 0.043\\ CR & 0.02 \end{array}\right]$$ (20)

$$\left[\begin{array}{cccccccc} & C_{1} & C_{2} & C_{3} & C_{4} & C_{5} & C_{6} & X_{9}\\ C_{1} & 1 & \frac{1}{6} & \frac{1}{6} & \frac{1}{6} & 1 & 1 & 0.047\\ C_{2} & 6 & 1 & 1 & 1 & 6 & 6 & 0.285\\ C_{3} & 6 & 1 & 1 & 1 & 6 & 6 & 0.285\\ C_{4} & 6 & 1 & 1 & 1 & 6 & 6 & 0.285\\ C_{5} & 1 & \frac{1}{6} & \frac{1}{6} & \frac{1}{6} & 1 & 1 & 0.047\\ C_{6} & 1 & \frac{1}{6} & \frac{1}{6} & \frac{1}{6} & 1 & 1 & 0.047\\ CR & 0.00 \end{array}\right]$$ (21)

$$\left[\begin{array}{cccccccc} & C_{1} & C_{2} & C_{3} & C_{4} & C_{5} & C_{6} & X_{10}\\ C_{1} & 1 & \frac{1}{3} & \frac{1}{3} & 3 & 1 & 1 & 0.111\\ C_{2} & 3 & 1 & 1 & 6 & 3 & 3 & 0.311\\ C_{3} & 3 & 1 & 1 & 6 & 3 & 3 & 0.311\\ C_{4} & \frac{1}{3} & \frac{1}{6} & \frac{1}{6} & 1 & \frac{1}{3} & \frac{1}{3} & 0.042\\ C_{5} & 1 & \frac{1}{3} & \frac{1}{3} & 3 & 1 & 1 & 0.111\\ C_{6} & 1 & \frac{1}{3} & \frac{1}{3} & 3 & 1 & 1 & 0.111\\ CR & 0.004 \end{array}\right]$$ (22)

The Eigenvectors corresponding to *F*_*1*_, *F*_*2*_, *F*_*3*_, *F*_*4*_, *F*_*5*_, *F*_*6*_, *F*_*7*_, *F*_*8*_, *F*_*9*_ and *F*_*10*_ are *X*_*1*_, *X*_*2*_, *X*_*3*_, *X*_*4*_, *X*_*5*_, *X*_*6*_, *X*_*7*_, *X*_*8*_, *X*_*9*_, and *X*_*10*_, respectively as shown in each matrix along with the *CR* values. *X*_*1*_ represents the Eigenvector corresponding to *F*_*1*_ criterion. Similarly, *X*_*2*_ represents the Eigenvector for the *F*_*2*_ criterion, *X*_*3*_ for *F*_*3*_ and so on. The *CR* value for calculating each eigen vector was verified to be less than 0.1.

#### Pairwise comparison of criteria with respect to alternatives

The ten features *F*_*1*_, *F*_*2*_, *F*_*3*_, *…*, *F*_*10*_ of the criteria are pairwise compared for all alternatives *C*_*1*_, *C*_*2*_, *C*_*3*_, *C*_*4*_, *C*_*5*_, and *C*_*6*_. The corresponding Eigenvectors for these alternatives are *X*_*11*_, *X*_*12*_, *X*_*13*_, *X*_*14*_, *X*_*15*_ and *X*_*16*_ as shown in matrices (23)-(28). The Eigenvectors for alternatives were calculated using similar calculations as we have done for criteria elements. The *CM*, *CI*, *λ*_*max*_ and the *CR* values for each matrix were calculated. The *CR* value for each eigenvector was checked and verified to be less than 0.1 for maintaining consistency among judgments. The result of comparisons for *C*_*1*_ and *C*_*2*_ alternatives with respect to each criterion are shown in matrix (23) and (24) respectively. The *CR* values satisfies the condition for the inconsistency in judgments, i.e., 0.09 and 0.04 both are less than 0.1; therefore, the judgments are pairwise consistent.

$$\left[\begin{array}{cccccccccccc} & F_{1} & F_{2} & F_{3} & F_{4} & F_{5} & F_{6} & F_{7} & F_{8} & F_{9} & F_{10} & X_{11}\\ F_{1} & 1 & \frac{1}{5} & 1 & 1 & 9 & 1 & 1 & 5 & 5 & 1 & 0.104\\ F_{2} & 5 & 1 & 1 & 1 & 9 & 5 & 5 & 7 & 7 & 5 & 0.290\\ F_{3} & 1 & 1 & 1 & 1 & 9 & 1 & 1 & 1 & 1 & 1 & 0.097\\ F_{4} & 1 & 1 & 1 & 1 & 9 & 1 & 1 & 1 & 1 & 1 & 0.097\\ F_{5} & \frac{1}{9} & \frac{1}{9} & \frac{1}{9} & \frac{1}{9} & 1 & \frac{1}{9} & \frac{1}{9} & \frac{1}{9} & \frac{1}{9} & \frac{1}{9} & 0.012\\ F_{6} & 1 & \frac{1}{5} & 1 & 1 & 9 & 1 & 1 & 5 & 5 & 1 & 0.104\\ F_{7} & 1 & \frac{1}{5} & 1 & 1 & 9 & 1 & 1 & 5 & 5 & 1 & 0.104\\ F_{8} & \frac{1}{5} & \frac{1}{7} & 1 & 1 & 9 & \frac{1}{5} & \frac{1}{5} & 1 & 1 & \frac{1}{5} & 0.041\\ F_{9} & \frac{1}{5} & \frac{1}{7} & 1 & 1 & 9 & \frac{1}{5} & \frac{1}{5} & 1 & 1 & \frac{1}{5} & 0.041\\ F_{10} & 1 & \frac{1}{5} & 1 & 1 & 9 & 1 & 1 & 5 & 5 & 1 & 0.104\\ CR & 0.09 \end{array}\right]$$ (23)

$$\left[\begin{array}{cccccccccccc} & F_{1} & F_{2} & F_{3} & F_{4} & F_{5} & F_{6} & F_{7} & F_{8} & F_{9} & F_{10} & X_{12}\\ F_{1} & 1 & 1 & 1 & 1 & 1 & 3 & 3 & \frac{1}{3} & 1 & 1 & 0.101\\ F_{2} & 1 & 1 & 1 & 1 & 1 & 3 & 3 & \frac{1}{3} & 1 & 1 & 0.101\\ F_{3} & 1 & 1 & 1 & 1 & 1 & 1 & 1 & 1 & 1 & 1 & 0.094\\ F_{4} & 1 & 1 & 1 & 1 & 1 & 1 & 1 & 1 & 1 & 1 & 0.094\\ F_{5} & 1 & 1 & 1 & 1 & 1 & 1 & 1 & 1 & 1 & 1 & 0.094\\ F_{6} & \frac{1}{3} & \frac{1}{3} & 1 & 1 & 1 & 1 & 1 & \frac{1}{4} & \frac{1}{3} & \frac{1}{3} & 0.054\\ F_{7} & \frac{1}{3} & \frac{1}{3} & 1 & 1 & 1 & 1 & 1 & \frac{1}{4} & \frac{1}{3} & \frac{1}{3} & 0.054\\ F_{8} & 3 & 3 & 1 & 1 & 1 & 4 & 4 & 1 & 3 & 3 & 0.201\\ F_{9} & 1 & 1 & 1 & 1 & 1 & 3 & 3 & \frac{1}{3} & 1 & 1 & 0.101\\ F_{10} & 1 & 1 & 1 & 1 & 1 & 3 & 3 & \frac{1}{3} & 1 & 1 & 0.101\\ CR & 0.04 \end{array}\right]$$ (24)

The pairwise consistency of the judgments with respect to each *C*_*3*_ and *C*_*4*_ alternatives were verified from the corresponding *CR* values. For each matrix the *CR* values fulfill the condition for pairwise consistency. i.e. *0*.*04≤0*.*1*and *0*.*07≤0*.*1* as shown in matrix (25) and (26).

$$\left[\begin{array}{cccccccccccc} & F_{1} & F_{2} & F_{3} & F_{4} & F_{5} & F_{6} & F_{7} & F_{8} & F_{9} & F_{10} & X_{13}\\ F_{1} & 1 & 1 & 1 & 1 & 1 & 5 & 3 & 1 & 1 & 1 & 0.122\\ F_{2} & 1 & 1 & 1 & 1 & 1 & 5 & 3 & 1 & 1 & 1 & 0.122\\ F_{3} & 1 & 1 & 1 & 1 & 1 & 1 & 1 & 1 & 1 & 1 & 0.093\\ F_{4} & 1 & 1 & 1 & 1 & 1 & 1 & 1 & 1 & 1 & 1 & 0.093\\ F_{5} & 1 & 1 & 1 & 1 & 1 & 1 & 1 & 1 & 1 & 1 & 0.093\\ F_{6} & \frac{1}{5} & \frac{1}{5} & 1 & 1 & 1 & 1 & \frac{1}{3} & \frac{1}{5} & \frac{1}{5} & \frac{1}{5} & 0.043\\ F_{7} & \frac{1}{3} & \frac{1}{3} & 1 & 1 & 1 & 3 & 1 & \frac{1}{3} & \frac{1}{3} & \frac{1}{3} & 0.063\\ F_{8} & 1 & 1 & 1 & 1 & 1 & 5 & 3 & 1 & 1 & 1 & 0.122\\ F_{9} & 1 & 1 & 1 & 1 & 1 & 5 & 3 & 1 & 1 & 1 & 0.122\\ F_{10} & 1 & 1 & 1 & 1 & 1 & 3 & 3 & 1 & 1 & 1 & 0.122\\ CR & 0.04 \end{array}\right]$$ (25)

$$\left[\begin{array}{cccccccccccc} & F_{1} & F_{2} & F_{3} & F_{4} & F_{5} & F_{6} & F_{7} & F_{8} & F_{9} & F_{10} & X_{14}\\ F_{1} & 1 & \frac{1}{3} & 9 & 9 & 9 & 9 & \frac{1}{5} & 1 & \frac{1}{5} & 1 & 0.088\\ F_{2} & 3 & 1 & 9 & 9 & 9 & 9 & \frac{1}{3} & 3 & \frac{1}{3} & 3 & 0.152\\ F_{3} & \frac{1}{9} & \frac{1}{9} & 1 & 1 & 1 & 1 & \frac{1}{9} & \frac{1}{9} & \frac{1}{9} & \frac{1}{9} & 0.015\\ F_{4} & \frac{1}{9} & \frac{1}{9} & 1 & 1 & 1 & 1 & \frac{1}{9} & \frac{1}{9} & \frac{1}{9} & \frac{1}{9} & 0.015\\ F_{5} & \frac{1}{9} & \frac{1}{9} & 1 & 1 & 1 & 1 & \frac{1}{9} & \frac{1}{9} & \frac{1}{9} & \frac{1}{9} & 0.015\\ F_{6} & \frac{1}{9} & \frac{1}{9} & 1 & 1 & 1 & 1 & \frac{1}{9} & \frac{1}{9} & \frac{1}{9} & \frac{1}{9} & 0.015\\ F_{7} & 5 & 3 & 9 & 9 & 9 & 9 & 1 & 5 & 1 & 5 & 0.261\\ F_{8} & 1 & \frac{1}{3} & 9 & 9 & 9 & 9 & \frac{1}{5} & 1 & \frac{1}{5} & 1 & 0.088\\ F_{9} & 5 & 3 & 9 & 9 & 9 & 9 & 1 & 5 & 1 & 5 & 0.261\\ F_{10} & 1 & \frac{1}{3} & 9 & 9 & 9 & 9 & \frac{1}{5} & 1 & \frac{1}{5} & 1 & 0.088\\ CR & 0.07 \end{array}\right]$$ (26)

$$\left[\begin{array}{cccccccccccc} & F_{1} & F_{2} & F_{3} & F_{4} & F_{5} & F_{6} & F_{7} & F_{8} & F_{9} & F_{10} & X_{15}\\ F_{1} & 1 & 7 & 9 & 1 & 9 & 3 & 3 & 4 & 7 & 4 & 0.268\\ F_{2} & \frac{1}{7} & 1 & 9 & 1 & 9 & \frac{1}{5} & \frac{1}{5} & \frac{1}{3} & 1 & \frac{1}{3} & 0.052\\ F_{3} & \frac{1}{9} & \frac{1}{9} & 1 & \frac{1}{9} & 1 & \frac{1}{9} & \frac{1}{9} & \frac{1}{9} & \frac{1}{9} & \frac{1}{9} & 0.011\\ F_{4} & 1 & 1 & 9 & 1 & 9 & 1 & 1 & 1 & 1 & 1 & 0.106\\ F_{5} & \frac{1}{9} & \frac{1}{9} & 1 & \frac{1}{9} & 1 & \frac{1}{9} & \frac{1}{9} & \frac{1}{9} & \frac{1}{9} & \frac{1}{9} & 0.011\\ F_{6} & \frac{1}{3} & 5 & 9 & 1 & 9 & 1 & 1 & 3 & 5 & 3 & 0.159\\ F_{7} & \frac{1}{3} & 5 & 9 & 1 & 9 & 1 & 1 & 3 & 3 & 3 & 0.159\\ F_{8} & \frac{1}{4} & 3 & 9 & 1 & 9 & \frac{1}{3} & \frac{1}{3} & 1 & 3 & 1 & 0.088\\ F_{9} & \frac{1}{7} & 1 & 9 & 1 & 9 & \frac{1}{5} & \frac{1}{5} & \frac{1}{3} & 1 & \frac{1}{3} & 0.052\\ F_{10} & \frac{1}{4} & 3 & 9 & 1 & 9 & \frac{1}{3} & \frac{1}{3} & 1 & 3 & 1 & 0.088\\ CR & 0.08 \end{array}\right]$$ (27)

$$\left[\begin{array}{cccccccccccc} & F_{1} & F_{2} & F_{3} & F_{4} & F_{5} & F_{6} & F_{7} & F_{8} & F_{9} & F_{10} & X_{16}\\ F_{1} & 1 & 9 & 9 & 1 & 9 & 1 & \frac{1}{5} & 1 & 1 & \frac{1}{3} & 0.090\\ F_{2} & \frac{1}{9} & 1 & 1 & \frac{1}{9} & 1 & \frac{1}{9} & \frac{1}{9} & \frac{1}{9} & \frac{1}{9} & \frac{1}{9} & 0.013\\ F_{3} & \frac{1}{9} & 1 & 1 & \frac{1}{9} & 1 & \frac{1}{9} & \frac{1}{9} & \frac{1}{9} & \frac{1}{9} & \frac{1}{9} & 0.013\\ F_{4} & 1 & 9 & 9 & 1 & 9 & 1 & 1 & 1 & 1 & 1 & 0.121\\ F_{5} & \frac{1}{9} & 1 & 1 & \frac{1}{9} & 1 & \frac{1}{9} & \frac{1}{9} & \frac{1}{9} & \frac{1}{9} & \frac{1}{9} & 0.013\\ F_{6} & 1 & 9 & 9 & 1 & 9 & 1 & \frac{1}{5} & 1 & 1 & \frac{1}{3} & 0.090\\ F_{7} & 7 & 9 & 9 & 1 & 9 & 5 & 1 & 5 & 5 & 3 & 0.294\\ F_{8} & 1 & 9 & 9 & 1 & 9 & 1 & \frac{1}{5} & 1 & 1 & \frac{1}{3} & 0.126\\ F_{9} & 1 & 9 & 9 & 1 & 9 & 1 & \frac{1}{5} & 1 & 1 & \frac{1}{3} & 0.090\\ F_{10} & 3 & 9 & 9 & 1 & 9 & 3 & \frac{1}{3} & 3 & 3 & 1 & 0.145\\ CR & 0.07 \end{array}\right]$$ (28)

The comparison matrices and the *CR* values for the last two alternatives, i.e. *C*_*5*_ and *C*_*6*_ are shown in matrix (27) and (28). The next step in the ANP model is the calculation of unweighted and weighted super-matrix.

#### Calculation of weighted super-matrix

**Fig 5:**
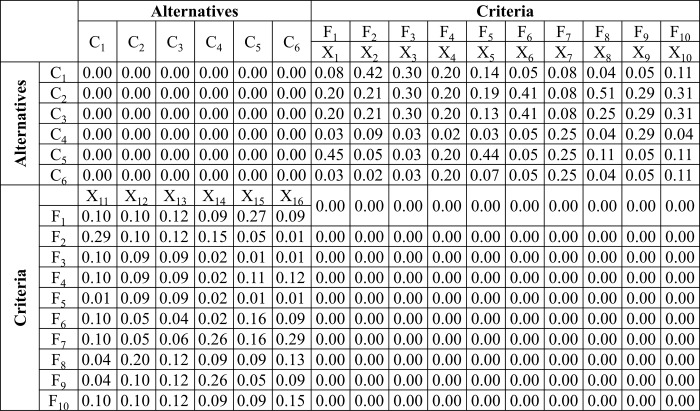
Calculation and representation of the eigenvectors in the weighted super-matrix.

The Eigenvectors (which show the weight of each criterion concerning each alternative and vice versa) were calculated as shown in matrices (13)-(28) are combined and represented in an unweighted super-matrix. Then, the unweighted super-matrix is revised to become column stochastic such that the sum of each column is equal to one. This action turns the matrix into a weighted super-matrix. The weighted super-matrix showing the comparison of the alternatives for criteria and vice versa is shown in Fig 5. The unweighted super-matrix is the same as the weighted super-matrix; However, the only difference between them is that the weighted super-matrix is column stochastic. Therefore, only the weighted super-matrix is shown in Fig 5. *X*_*1*_, *X*_*2*_, *X*_*3*_, *X*_*4*_, *X*_*5*_, *X*_*6*_, *X*_*7*_, *X*_*8*_, *X*_*9*_, and *X*_*10*_ are the Eigenvectors corresponding to *F*_*1*_, *F*_*2*_, *F*_*3*_, *F*_*4*_, *F*_*5*_, *F*_*6*_ *F*_*7*_, *F*_*8*_, *F*_*9*_, *F*_*10*_ represent the priority values of the criteria (features) for each alternative. Similarly, *X*_*11*_, *X*_*12*_, *X*_*13*_, *X*_*14*_, *X*_*15*_, *X*_*16*_ are the Eigenvectors corresponding to *C*_*1*_, *C*_*2*_, *C*_*3*_, *C*_*4*_, *C*_*5*_, and *C*_*6*_ represent the priority values of the alternatives (controllers) concerning each feature. To obtain the final stable weights of the alternatives the next step in the ANP model is the calculation of limit super-matrix.

#### Calculation of limit super-matrix

**Fig 6:**
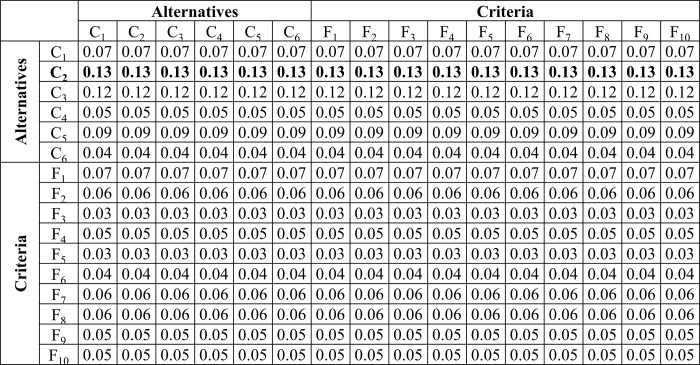
Limit super-matrix.

**Fig 7:**
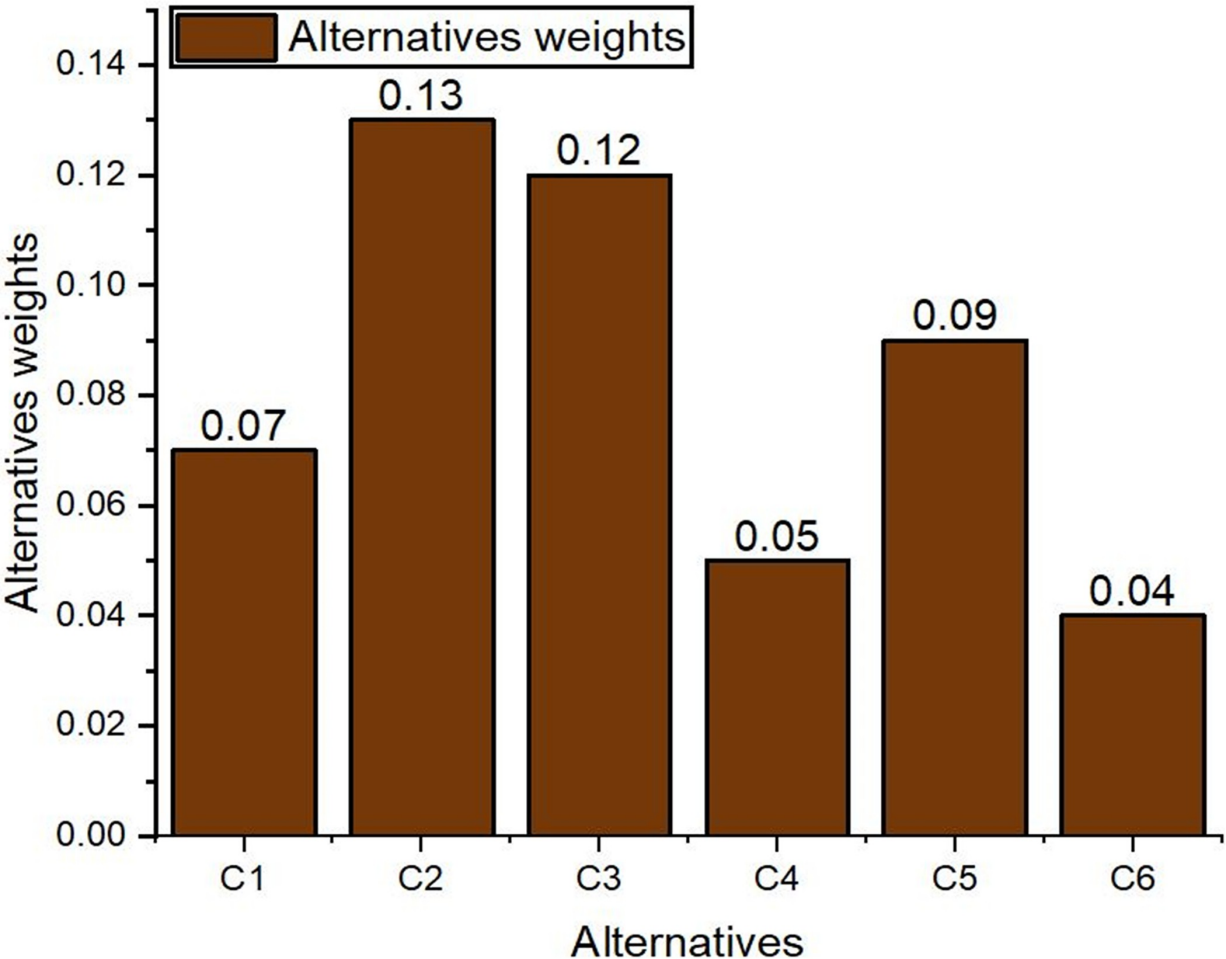
Alternatives weights from the limit super-matrix.

The weighted super-matrix was processed by raising it to a larger power until it was converged to a matrix with stable values. The stable matrix is called a limit super-matrix. The limit matrix shows the weights of the alternatives and the criteria, i.e., the final prioritized values. The limit matrix is the resultant matrix that contains the final weights measured against each element in the criteria and alternative clusters. It was obtained from the weighted super-matrix where the values were raised to the power of *2k* to obtain same value for each row, where *k* represents any random number. The limit super-matrix summarizes the pairwise comparisons of all matrices. It also shows the indirect relationship between components. Fig 6 represents the limit super-matrix, where higher value represents the standing alternative. Final stable weights of all alternatives from Fig 6 are shown in Fig 7. It illustrates that *C*_*2*_ have the highest weights; therefore, this is the most suitable controller. The next suitable controllers are *C*_*3*_, *C*_*5*_, *C*_*1*_, *C*_*4*_, and *C*_*6*_ according to their final weights calculated from the limit super-matrix. Fig 7 shows the final alternatives weights and the stability of the results was verified through the limit matrix. According to the results, *C*_*2*_ have high-weight value and therefore the SDN controller corresponding to it is the proposed controller with ANP model. Further, the performance of the proposed controller was evaluated with simulations and compared with the controller proposed with AHP model. In the next section the results of the simulations for both controllers are discussed.

## Experimental setup design

**Fig 8:**
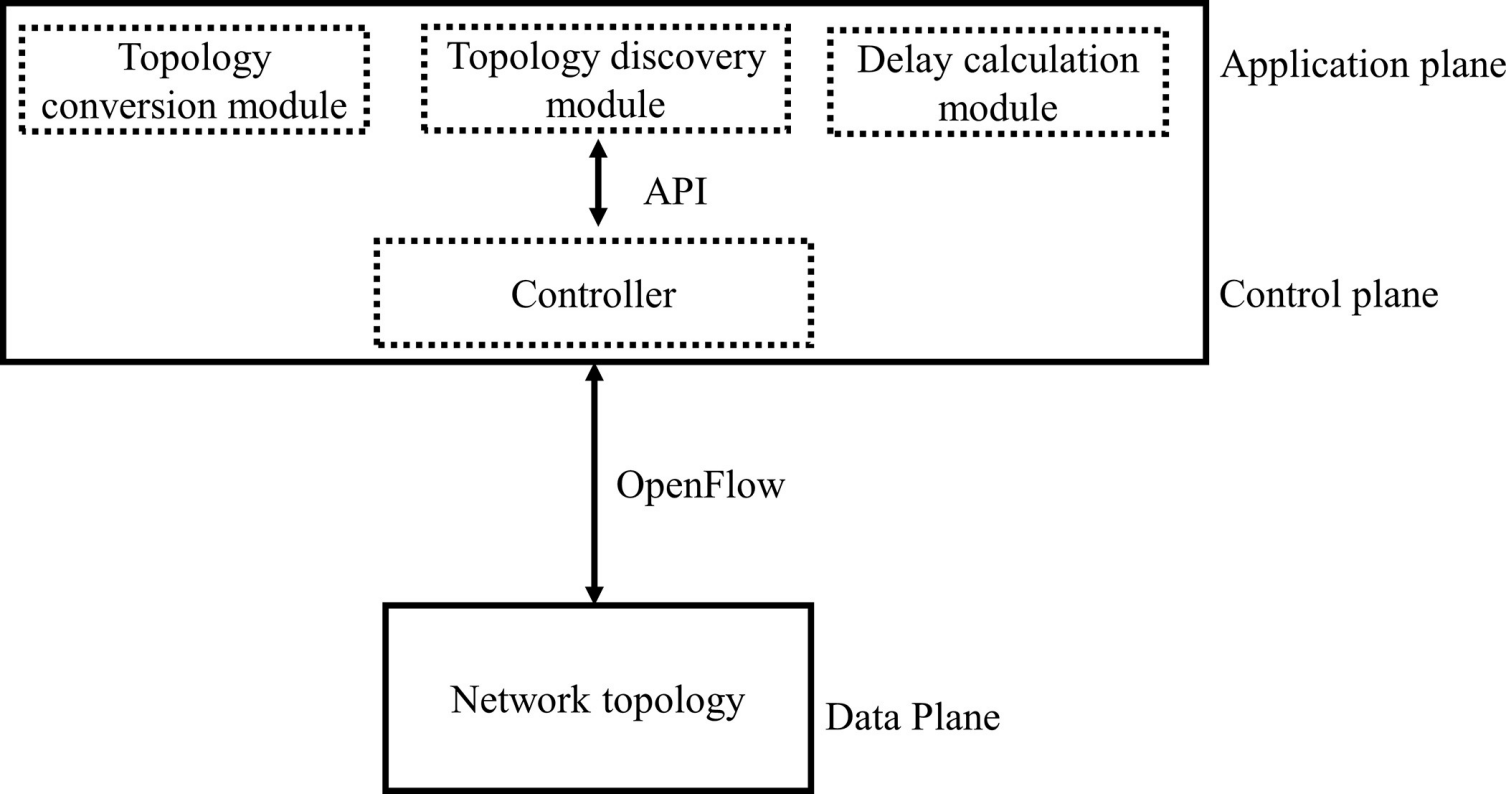
Experimental framework.

Fig 8 shows the experimental framework used for the performance evaluation. The performance analysis was conducted for *C*_*2*_ controller computed using the proposed approach, i.e. ANP. A performance comparison was made with the controller (RYU) calculated through AHP. First, the network topologies considered for experiments were converted to Mininet environment. The source and destination pairs of routers were selected in each topology and shortest path discovery time was calculated. i.e. the time a controller took for discovering the shortest path. After this, two hosts were attached with source and destination. Furthermore, the delay was computed for the request and response time taken by a controller for that path in the normal and a traffic generation scenario. Likewise, the throughput and CPU utilization were recorded for each controller. The experiments were performed for both controllers. Experimental scenario design and performance evaluation are discussed below;

### Network topologies

The standard network topologies, i.e. Abilene, European reference network (ER_Net) [55], USA backbone IP network (US_Net) [56] and open Science, scholarship and services exchange (OS3E) [57], were considered for analysis. The topologies were represented as graph *G* = (*V*,*E*), where *V* represents the vertices and *E* represents the edges of the topology. The information provided in graphical form was used to build the topologies. Besides this, two large scale topologies named as B_1 and B_2 were created with Brite topology generator. A well-known Waxman [58] model was used for connecting the routers. The routers distribution in the plane is based on Eq (29);

$$P\left(e,v\right)=\beta exp^{\frac{-d\left(e,v\right)}{M\alpha }}$$ (29)

In Eq (29) *α* > 0 and *β* ≤ 1. Herein, the d shows the distance between *e* and *v*. The *M* shows maximum the distance of any two given routers between them. The links and edges are related to *α* and *β*. The link number is directly proportional with α and distance between edges increases when the *β* is incremented. The output file containing information about the topology generation model, nodes and edges number was parsed with Fast Network Simulation Setup (FNSS) [59]. FNSS core python library and adapters were used to export the Brite generated output file to Mininet. Table 6 shows the topologies evaluated for performance analysis.

**Table 6:** Real-world and Brite topologies.

Topology	Nodes	Edges
Abilene	11	14
ER_Net	37	57
US_Net	24	42
OS3E	34	41
B_1	100	200
B_2	200	400

### Mininet

Mininet Python API was used for emulating the network topologies on the two controllers computed with proposed and AHP approach. This network emulator has been used widely for prototyping SDN-based experiments. The Mininet latest version 2.3.0d1 and an OpenvSwitch (OVS) version of 2.5.4 was installed in Ubuntu 16.04 LTS. Further, the Xming server was started to generate and visualize traffic between the source and destination hosts.

### Experiment example

**Fig 9:**
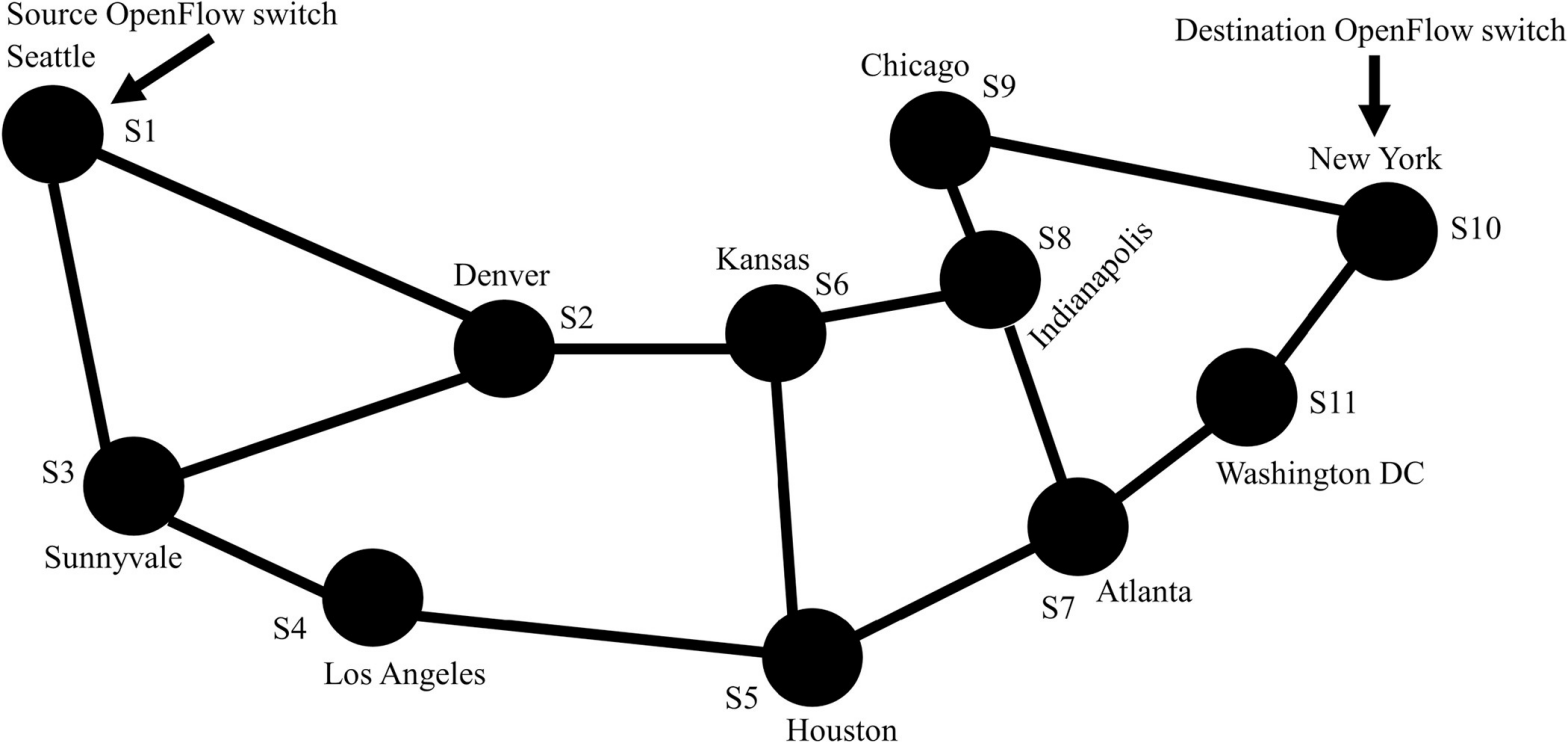
Path calculation example between source and destination OpenFlow switches using Abilene topology.

A simple example herein explains how the experiment was performed for a real-world topology Abilene as shown in Fig 9. First, the topology was converted and emulated in Mininet using the topology conversion module. Then source *S*_*s*_ (Seattle) and *D*_*s*_ destination (New York) are chosen and the shortest path between them is computed using the Dijkstra Algorithm [60]. The algorithm returns the following route.

$$Seattle\left(S_{1}\right)\to Denver\left(S_{2}\right)\to Kansas\left(S_{6}\right)\to Indianapolis\left(S_{8}\right)\to Chicago\left(S_{9}\right)\to New\;York\left(S_{10}\right)$$

In case multiple routes were returned by the algorithm, then one of them was chosen randomly. After that those hosts (H_1_ and H_2_) were attached with source and destination OpenFlow switches (Seattle and New York City). Eqs (30) and (31) represent the shortest path and all possible set of paths returned by Dijkstra algorithm between source *S*_*s*_ and destination *D*_*s*_. For network topology with a graph *G* = (*V*,*E*), where *V* and *E* denote the vertices and edges. In Eq (31), The *P* represents shortest route between *S*_*s*_ and *D*_*s*_, where *P* = *D*(*PS*_*s*_,*PD*_*s*_) denotes hop count distance from *S*_*s*_ to *D*_*s*_.

$$P(S_{s},D_{s})=shortest-path(S_{s},D_{s})$$ (30)

$$P_{set}=\{P|\forall S_{s},D_{s}\in V:P=D(PS_{s},{PD}_{s})\}$$ (31)

## Performance evaluation

### Topology discovery

**Fig 10:**
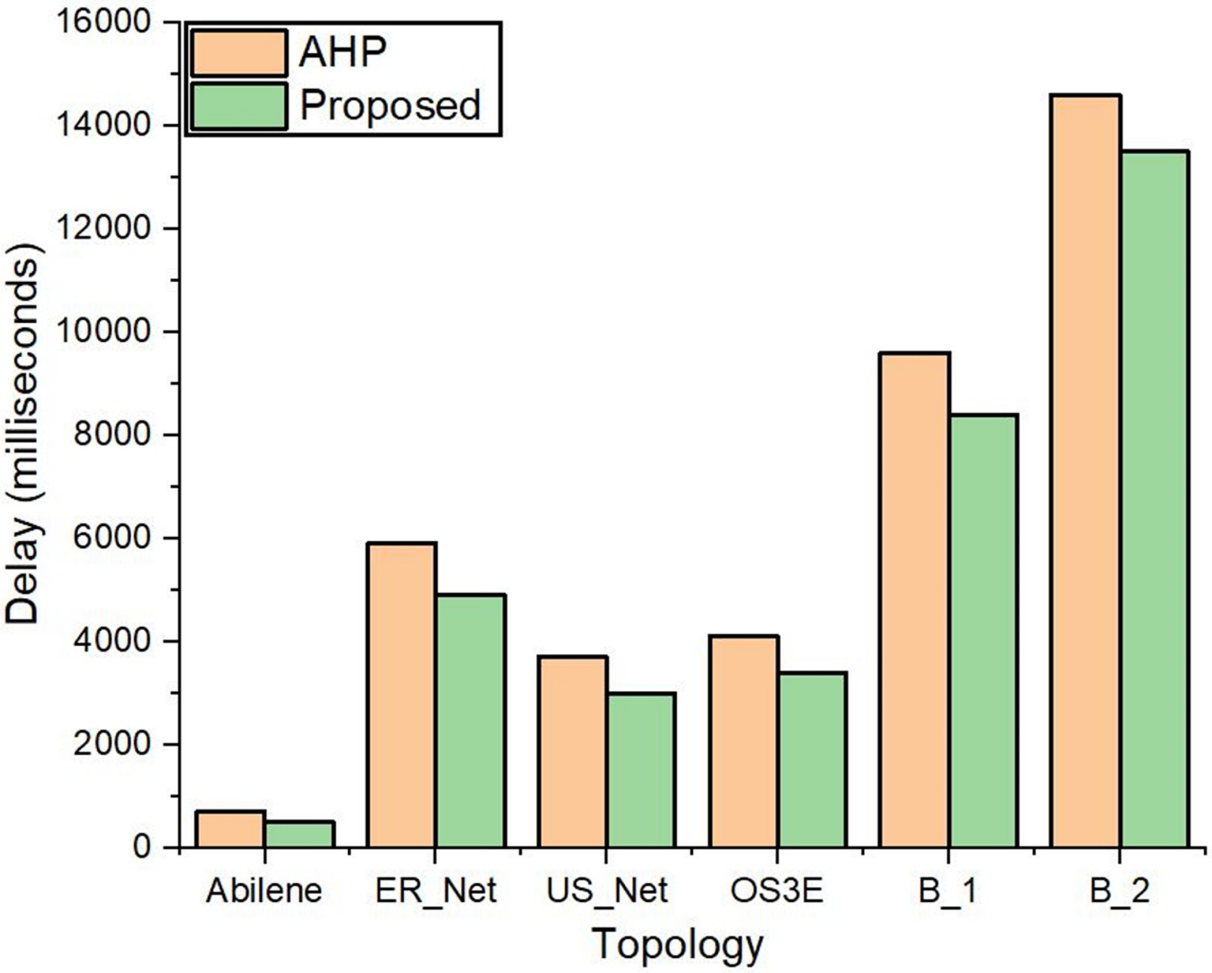
Delay in path discovery process.

The discovery time is the time taken by a controller to discover the shortest route between source and destination in each topology. Therefore, it is the time taken by the controller before pushing flow entries into the OpenFlow switches. The discovery time for the two controllers have been computed for the six topologies as shown in Fig 10. The results indicate that the proposed controller has a less topology discovery time than the controller computed with the AHP approach. The topologies generated with Brite have a more substantial topology discovery than real-world Internet topologies. In the small topologies with lesser number of nodes such as Abilene, US_Net, OS3E, and ER_Net the shortest path length is 7% of the total nodes. However, it is up to 12% in Brite generated topologies (B_1 and B_2) and therefore the discovery time for these topologies is large. The proposed controller has fast state synchronization capability, i.e. the way it stores and handles the information of the underlying topology. The percentage decrease in topology discovery time with proposed controller is 28.57% in Abilene, 18.91% in US_Net, 17.07% in OS3E, 16.94% in ER_Net, 12.5% in B_1 and 7.53% in B_2 topology.

### Delay measurement

The delay was measured for each topology by selecting the source and destination OpenFlow switches. The shortest path between source and destination was calculated. Flow entries were pushed in the route via REST API after the hosts were attached with source and destination OpenFlow switches.

**Fig 11:**
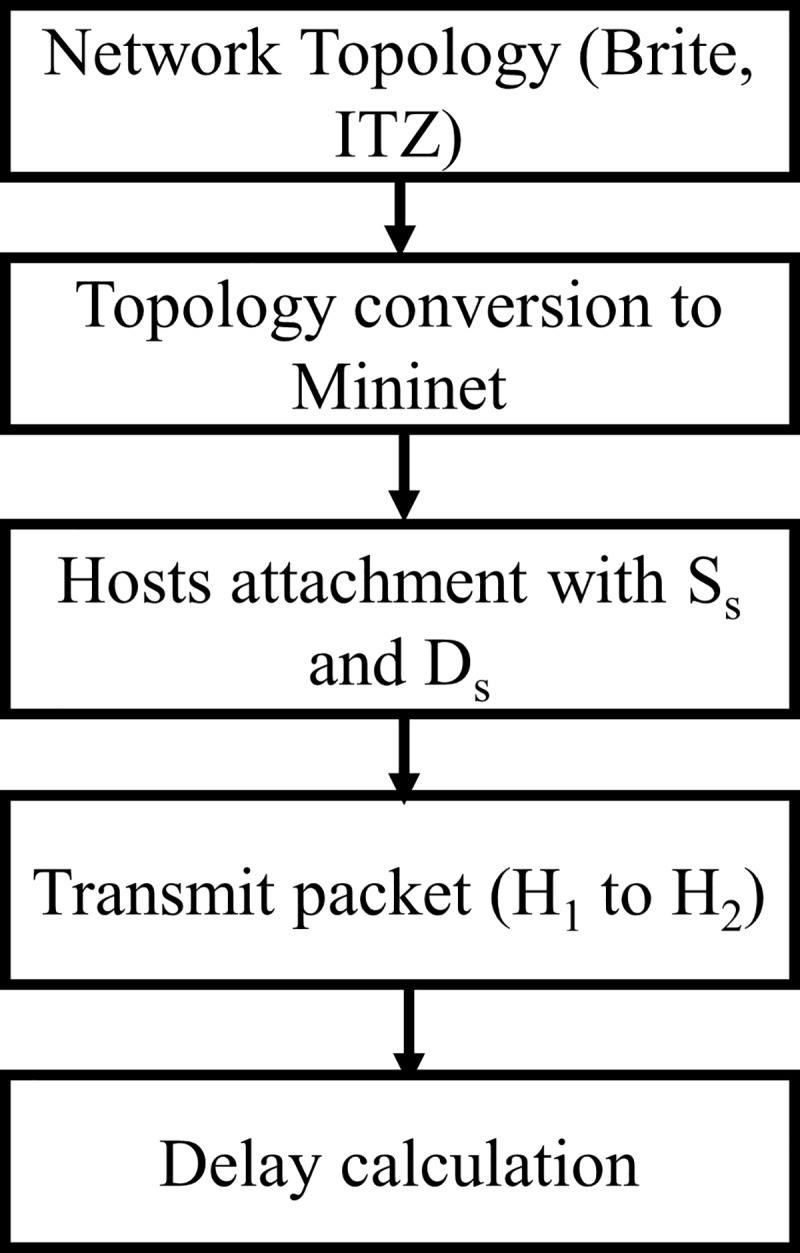
Delay measurement process.

**Fig 12:**
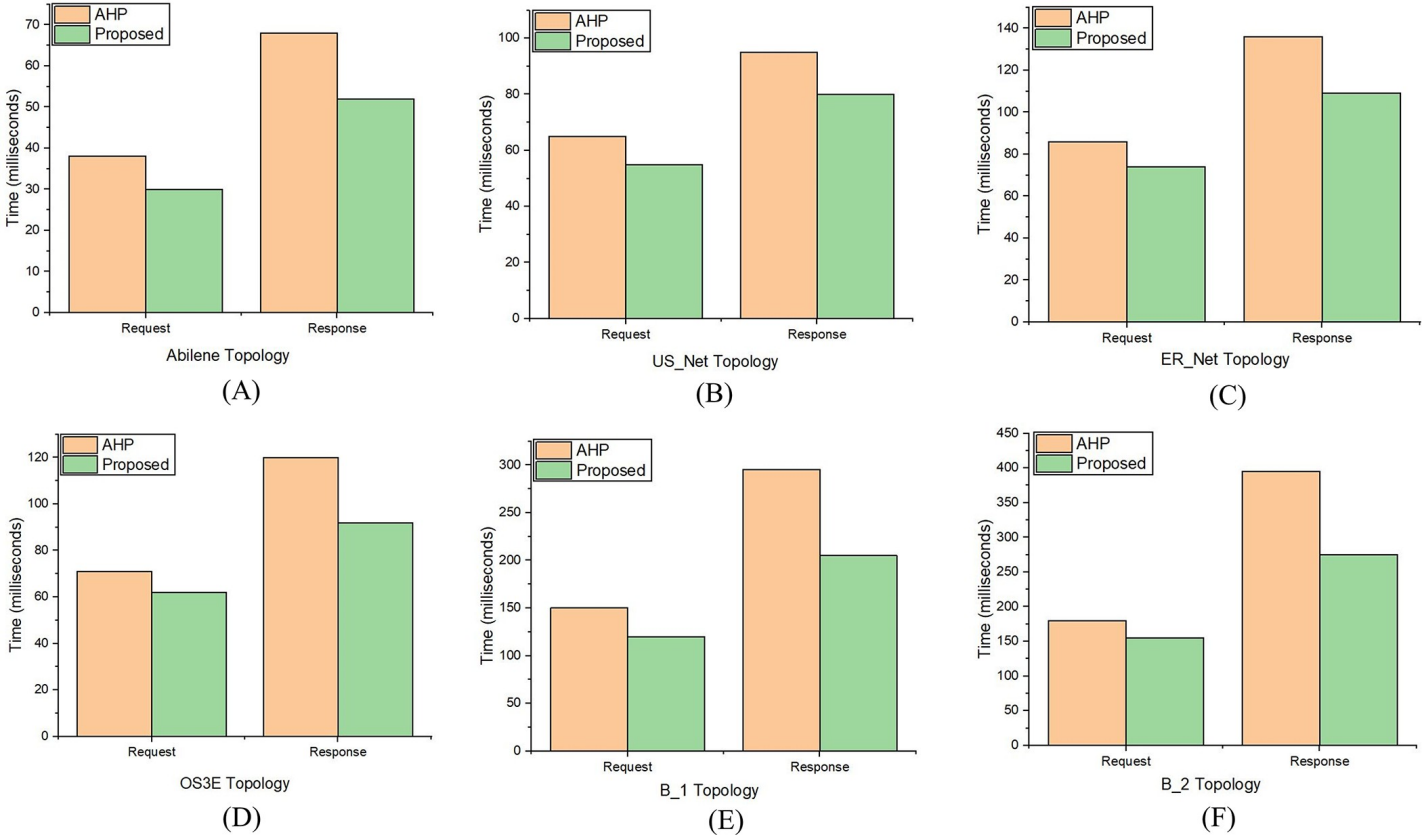
Request and response time measurement for (A) Abilene topology (B) US_Net topology (C) ER_Net topology (D) OS3E topology (E) B_1 topology (F) B_2 topology.

The delay for an IP packet was measured by statically setting the address resolution protocol (ARP) cache, therefore, the ARP requests and responses were not sent in the network topology. An IP packet was then sent from the source to destination host and the delay was recorded. The request and response time of each packet was measured with tcpdump. The procedure is shown in Fig 11. The Dijkstra's algorithm [60], has a complexity of *O(|V|+|E| log |V|)*. The graph size *(V × E)* and time complexity for the Dijkstra’s algorithm are directly proportional. Therefore, the number of search space increases as the distance between the S_s_ and D_s_ increase [61]. The delay calculated for each topology is shown in Fig 12A–12F. The topologies generated with Brite has more delay due to the complexity of the structure and increase in distance between *S*_*s*_ and *D*_*s*_. It is evident from Fig 12E and 12F where the request and response time gap is larger than the other topologies. The average number of nodes between S_s_ and D_s_ increases from 7% in the real-world Internet topologies to 12% in the Brite topologies. Therefore, the number of search space increase for Dijkstra Algorithm resulting in increased delay. The delay for the proposed controller is less than the controller selected via AHP. The percentage decrease is 44.18%, 41.37%, 38%, 30%, 26.66% and 16.66% for B_2, B_1, OS3E, ER_Net, Abilene and US_Net topologies respectively. It is due to the less time of the flow rules insertion during the path setup via the fast-responsive REST API of the proposed controller. Consequently, the delay of the proposed controller is less than the AHP controller.

### Delay measurement with traffic load

**Fig 13:**
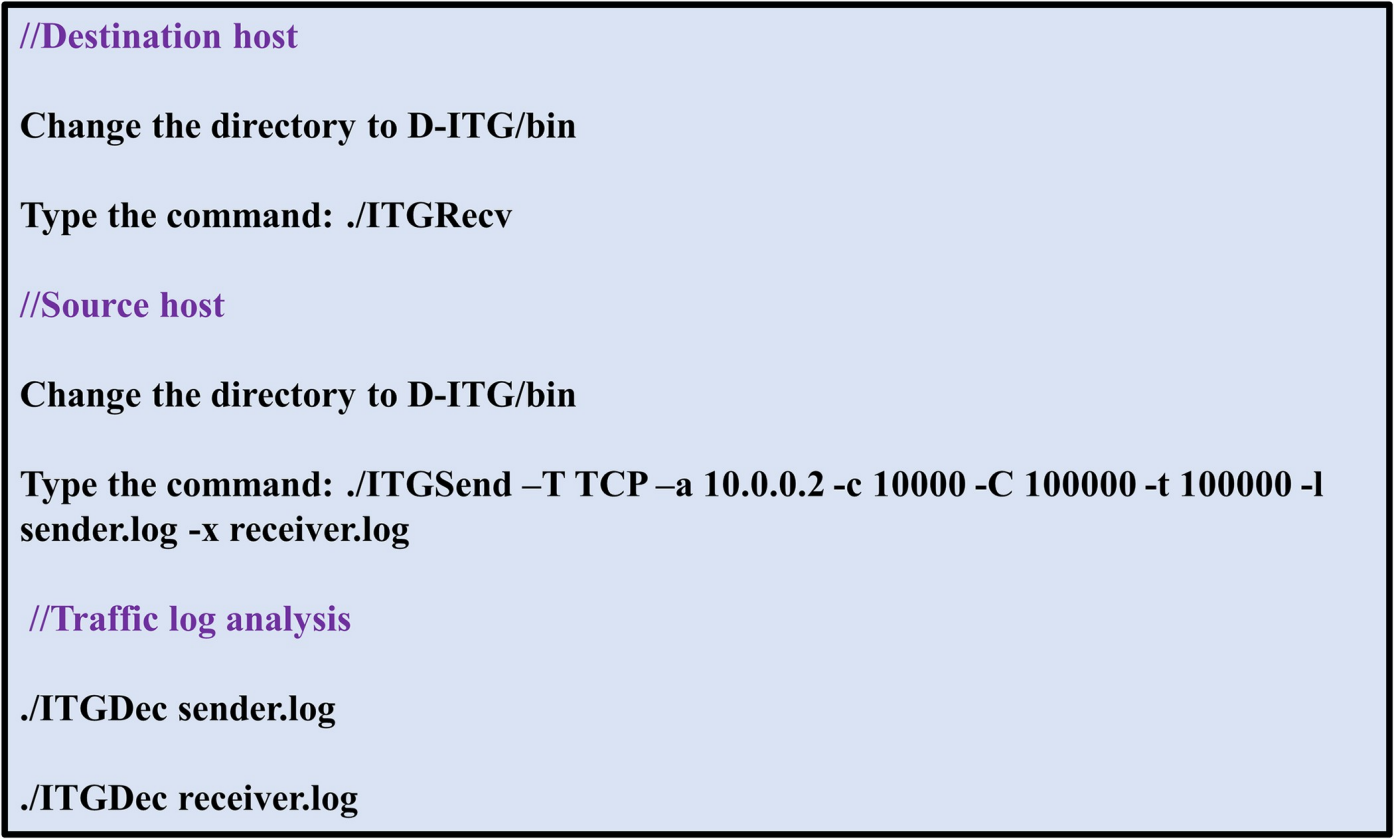
Traffic generation methodology between source and destination hosts.

**Fig 14:**
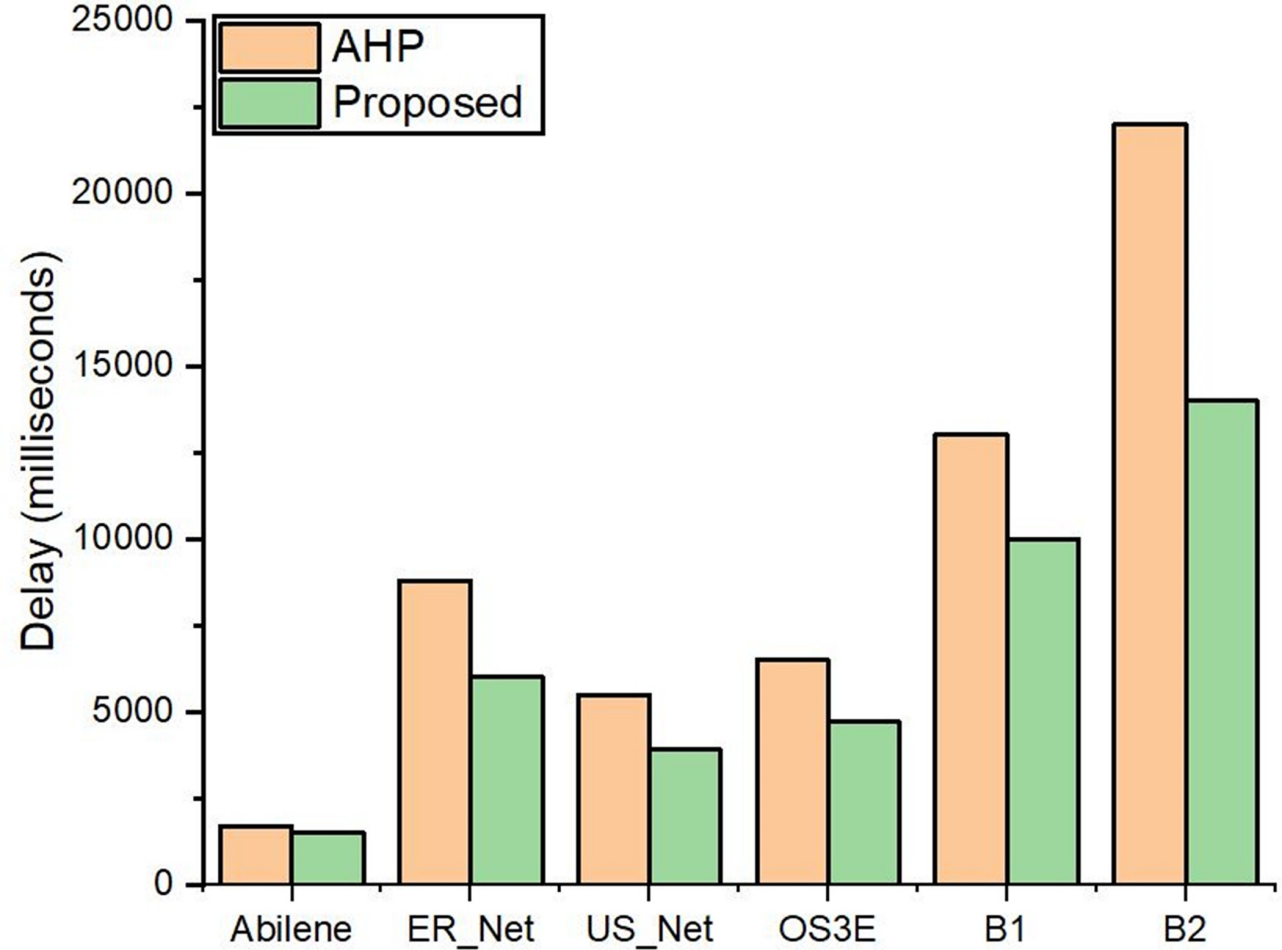
Delay calculated by putting the controllers to traffic load.

The distributed Internet traffic generator (D-ITG) [62] has been used for traffic generation between the source and destination nodes in each topology. The procedure for generating traffic between source and destination hosts is described in Fig 13. A listening socket is opened for transmission control protocol (TCP) traffic coming from the other nodes through ITGRecv on the destination host (H_2_). On the source host (H_1_), ITGSend is used to send TCP traffic having a payload of 10000 bytes for 100 seconds (sec) constantly with the rate of 100000 packets/sec, to the destination IP address. The experiment performed for each topology was repeated ten times, and the average results for delay are shown in Fig 14.

The results indicate that delay calculated with high traffic generation between source and destination hosts for the proposed controller is lesser than AHP controller. It is due to the delay reduction in topology discovery and flow insertion for the optimum controller. The results for the large-scale B_1 and B_2 topologies have a long path discovery, and path setup delays due to their complex structure and increased hop count between source and destination hosts. This results in the delay increase for these topologies as compared to real-world internet topologies.

### Throughput

**Fig 15:**
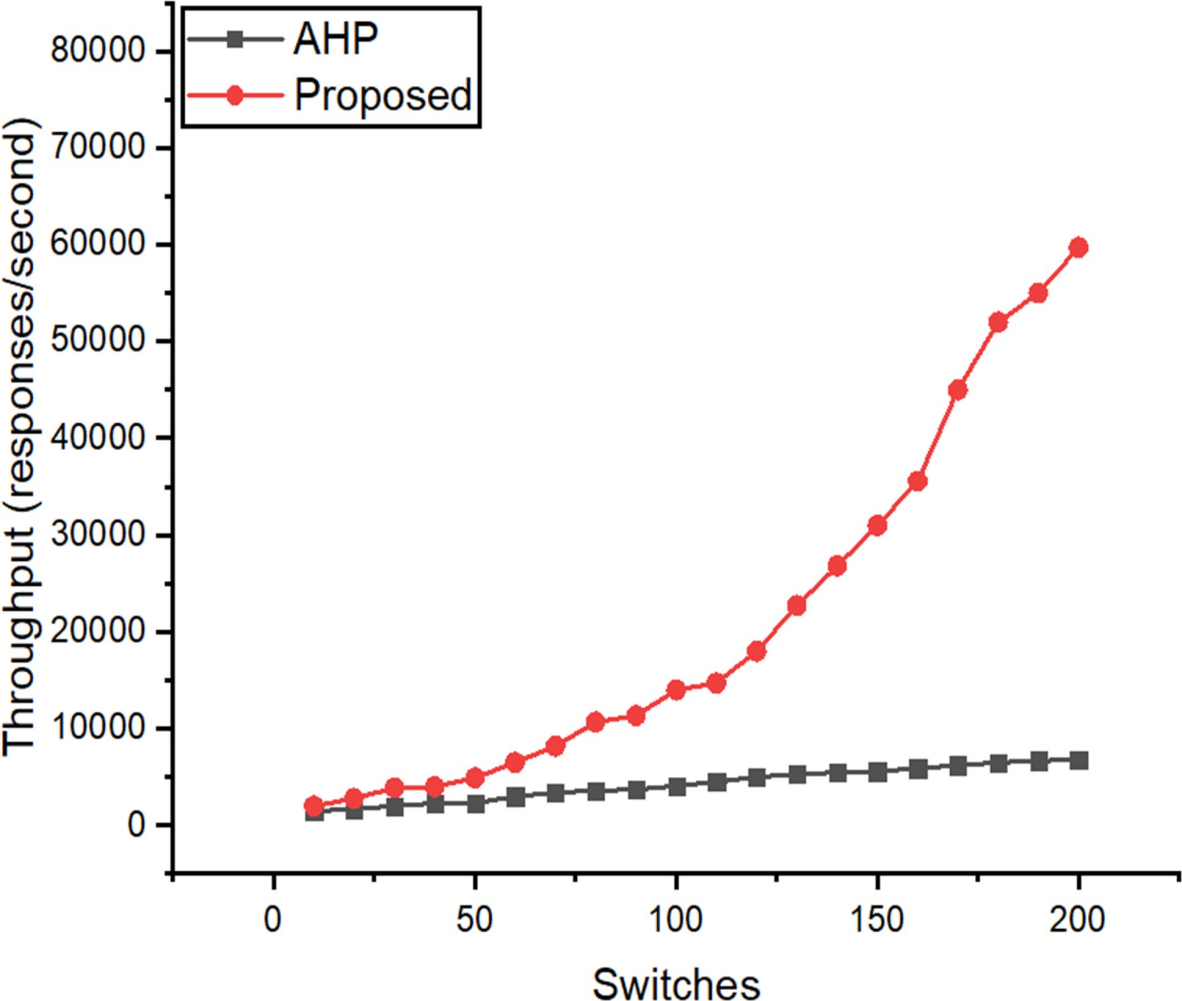
Throughput calculated with respect to increasing the number of switches.

The throughput was computed using Cbench by sending PACKET_IN messages to the controller and calculating the number of PACKET_OUT (responses/second). Herein, the number of MACs emulated per switch were kept in 2000. The number of switches was varied up to 200, and each test was performed ten times. The average results show that the throughput of the proposed controller does not degrade and has a quick start as compared to the controller proposed with AHP. The result for the performance is shown in Fig 15.

### CPU utilization

**Fig 16:**
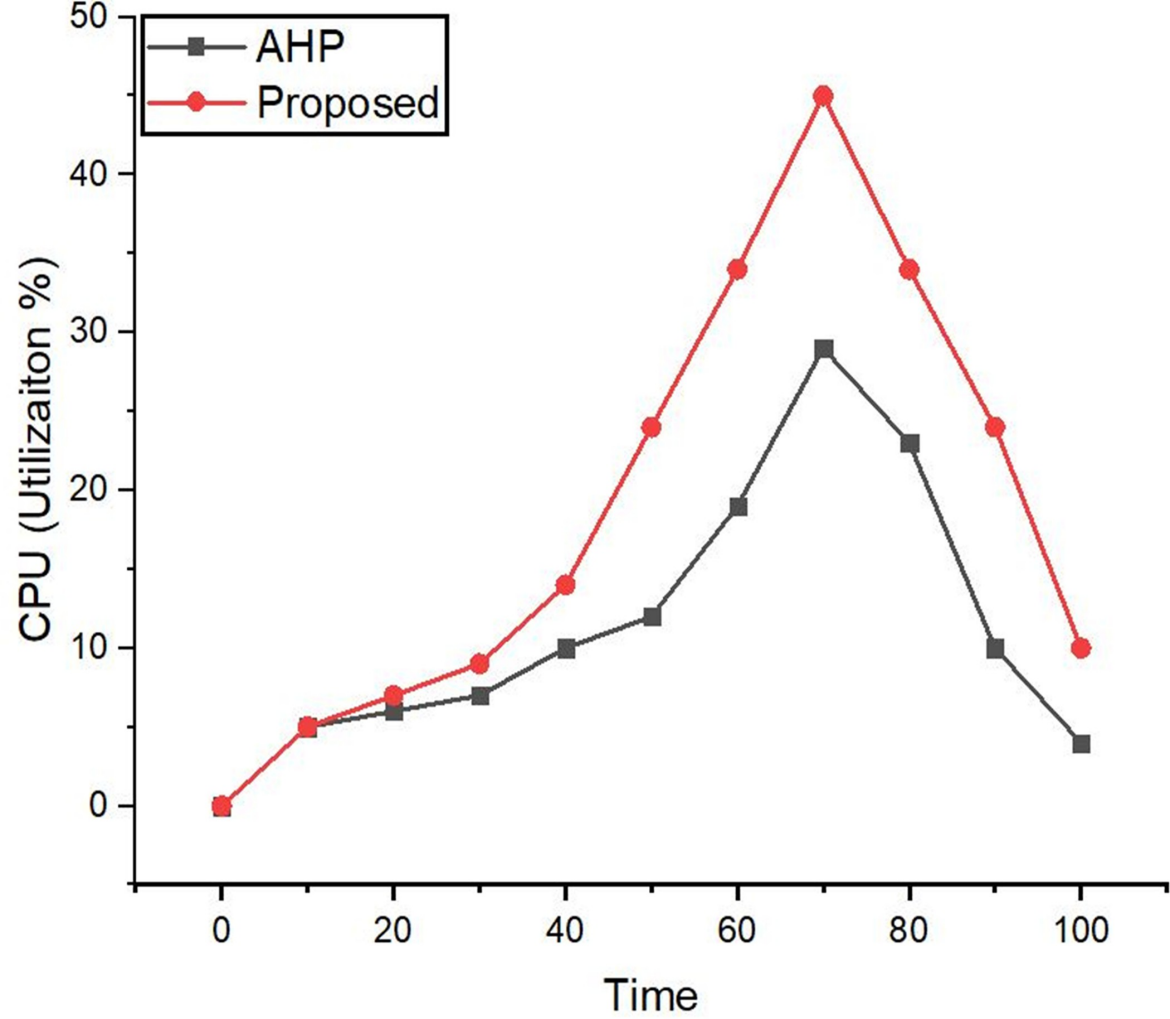
CPU utilization (percentage).

The CPU utilization was measured with sysbench [63] tool while testing both controllers, i.e. selected via AHP and the proposed approach during the traffic generation. Fig 16 shows the results for CPU utilization at 20 seconds intervals. The graph shows that during the peak usage the utilization does not surpass from 30% and 45% for a controller with AHP and the proposed approach. During the normal condition, this utilization does not exceed 19% for the controller proposed with AHP and 26% for the controller with the proposed method.

## Conclusion and future work

The objective of this study was to select the optimum SDN controller regarding its features and the performance analysis of the controller in real-world and Brite network topologies. Because the controller selection process was based on multiple features, which include platform support, southbound interface, northbound interface, modularity, and productivity, it was considered to be an MCDM problem. Therefore, the ANP approach was used to solve this problem. The objectives were identified first, and criteria parameters were established based on which the proposed controller was computed using ANP model. Next, a pairwise comparison matrix was created to compare every element in the criterion cluster with every alternative in the alternative cluster, and vice versa. The final resultant matrix, known as a limit matrix, prioritizes the alternatives. Thus, controller with high-priority value was proposed for further quantitative analysis. The results from the limit super-matrix showed that *C*_*2*_ controller provides the optimum features, therefore its performance was validated in Mininet.

To verify the performance of the two feature-based optimum controllers, i.e. proposed approach and AHP, a quantitative comparison of the two controllers was performed by measuring the QoS of the two controllers, such as topology discovery time, delay, throughput, and CPU utilization. The experimental results validated through Mininet showed that *C*_*2*_ outperforms *C*_*5*_ for both the Internet and Brite topologies. Through the proposed methodology, we selected the controller with high-priority value with respect to its supporting features compared to other controllers considered in the experiment. In this case, *C*_*2*_ is the optimum controller because it fulfils the maximum required features and it is also quantitatively better than AHP based controller. The ANP can also be used for the optimization of criteria parameters therefore, in the future, we want to see its results for criteria optimization problems in SDN.
